# Macropinocytosis inhibition attenuates profibrotic responses in lung fibroblasts and pulmonary fibrosis models

**DOI:** 10.1172/JCI197651

**Published:** 2026-04-30

**Authors:** Ivan O. Rosas, Aaron K. McDowell-Sanchez, Santiago Sanchez, Juan D. Cala-Garcia, Alan R. Waich Cohen, Elisa Ruiz-Echartea, Scott A. Ochsner, Daniel C. Kraushaar, Lindsay J. Celada, Dandan Sun, Francesca Polverino, Cristian Coarfa, Neil J. McKenna, Konstantin Tsoyi

**Affiliations:** 1 Section of Pulmonary, Critical Care, and Sleep Medicine, Department of Medicine,; 2 Department of Molecular and Cellular Biology, and; 3 Genomic and RNA Profiling Core, Baylor College of Medicine, Houston, Texas, USA.; 4 Department of Neurology, Pittsburgh Institute for Neurodegenerative Disorders, University of Pittsburgh, Pittsburgh, Pennsylvania, USA.; 5 Veterans Affairs Pittsburgh Health Care System, Geriatric Research, Educational and Clinical Center, Pittsburgh, Pennsylvania, USA.; 6 Division of Pulmonary, Critical Care and Sleep Medicine, Department of Medicine, University of Maryland School of Medicine, Baltimore, Maryland, USA.

**Keywords:** Cell biology, Pulmonology, Fibrosis

## Abstract

Idiopathic pulmonary fibrosis (IPF) is a devastating chronic lung disorder with limited treatment options. Macropinocytosis is one of the key cellular processes involved in nutrient consumption from the extracellular environment under stress conditions. Here, we studied the role of macropinocytosis in experimental pulmonary fibrosis models. We found that macropinocytosis is increased in human lung fibroblasts (HLFs) derived from patients with IPF. The inhibition of macropinocytosis with 5-(n-ethyl-n-isopropyl)-amiloride (EIPA) inhibited profibrotic responses in IPF-derived and TGF-β1–stimulated HLFs and reduced pulmonary fibrosis in bleomycin-injured (Bleo-injured) mice. EIPA exerted its antifibrotic effects by regulating amino acid uptake, mammalian target of rapamycin complex 1 (mTORC1) activation and mesenchyme homeobox1 (*MEOX1*) expression in activated HLFs. Fittingly, genetic inhibition of macropinocytosis also ameliorated lung fibroblast activation and pulmonary fibrosis in mice. Using IPF-derived precision cut lung slices (PCLSs), we observed robust repression of profibrotic gene expression programs in EIPA-treated PCLSs across different fibroblast subpopulations. Finally, we found that imipramine (Imi), a tricyclic antidepressant approved by the FDA, effectively inhibited macropinocytosis and ameliorated profibrotic responses in lung fibroblasts, Bleo-injured mice, and IPF-derived PCLSs. Taken together, our results suggest that macropinocytosis inhibition can be considered as a potential therapeutic strategy to treat pulmonary fibrosis.

## Abstract 2

Activated lung fibroblasts are the hallmark of pulmonary fibrosis. We found that macropinocytosis contributes to the profibrotic responses in activated lung fibroblasts. Thus its inhibition demonstrated potent anti-fibrotic effects in experimental models of pulmonary fibrosis

## Introduction

Idiopathic pulmonary fibrosis (IPF) is a chronic, progressive, fibroproliferative interstitial lung disease of enigmatic etiology (1). IPF affects approximately 500 per 100,000 adults over the age of 65 in the United States alone and is increasing in prevalence, leading to rising rates of hospital admissions and deaths (2). Currently, there are two FDA-approved therapies to treat IPF, nintedanib and pirfenidone, both of which can reduce decline in lung function, decrease number of acute exacerbations, and improve survival (3, 4). However, neither of these drugs can stop or reverse disease progression. Thus, there is an unmet need to determine the molecular mechanisms underlying the pathogenesis of IPF to enable the development of new and effective therapeutics.

Fibroblasts/myofibroblasts are among the primary cell types responsible for the accumulation of extracellular matrix (ECM) and organ remodeling in fibrotic disorders (5). Fibroblasts can be activated by pathological stimuli, such as TGF-β and substrate stiffness, prompting these cells to express increased α-smooth muscle actin (α-SMA) stress fibers, deposit ECM proteins, display increased contractility and invasiveness, and develop resistance to proapoptotic stimuli (e.g., Fas ligand) (6, 7). Thus, targeting these processes in fibroblasts represents an opportunity for therapeutic intervention in fibrotic disorders.

Macropinocytosis is an actin-dependent, but clathrin-independent, endocytic process that mediates the nonselective internalization of extracellular materials such as proteins, cell debris, or viruses (8). Macropinosomes are formed when cell membrane ruffles curve to form circular cup-shaped structures, which eventually seal and pinch off to form large vesicles (greater than 0.2 μm in diameter) (8). Macropinocytosis can function as a means of nutrient acquisition in mammalian cells, which, in turn, can greatly influence cell survival and proliferation (9, 10). Multiple reports also highlight the key role for macropinocytosis in various malignant disorders by providing essential nutrients to support the proliferation and metastasis of tumor cells under nutrient poor conditions (8, 11). However, the role of macropinocytosis in organ fibrosis remains unclear.

Here, we report that macropinocytosis inhibition exerted potent antifibrotic effects in activated lung fibroblasts by regulating amino acid (AA) uptake and mammalian target of rapamycin complex 1 (mTORC1) activation. We also found that imipramine (Imi), a clinically used tricyclic antidepressant, inhibited macropinocytosis in IPF-derived lung fibroblasts and ameliorated experimental pulmonary fibrosis in ex vivo and in vivo models.

## Results

### Macropinocytosis inhibition attenuates profibrotic responses in activated HLFs.

**Figure 1:**
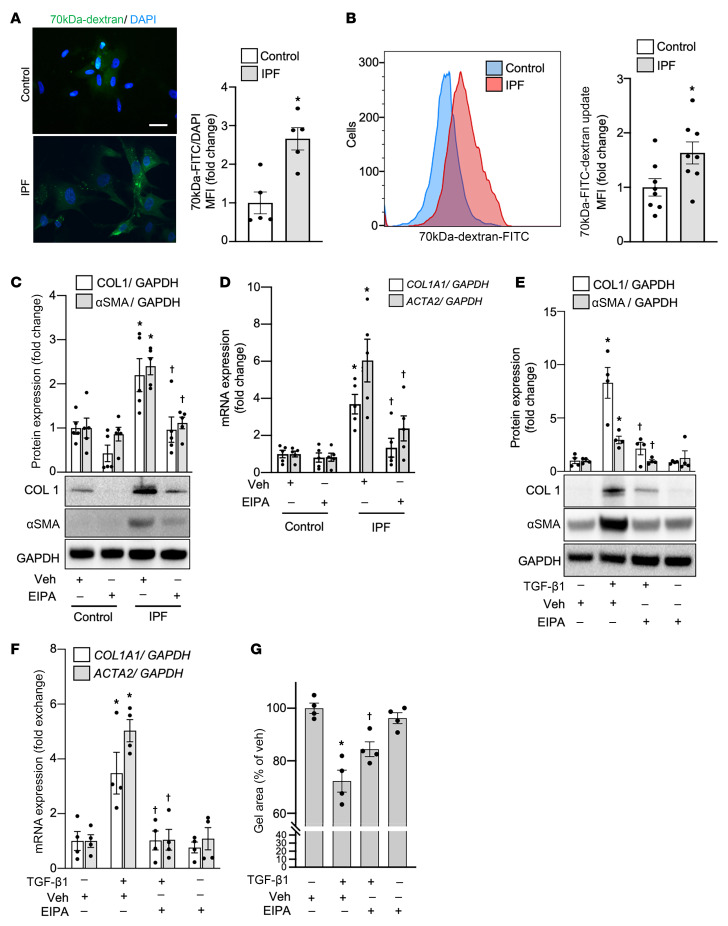
Macropinocytosis is elevated in IPF-derived lung fibroblasts and its inhibition regulates profibrotic responses. (**A** and **B**) Control and IPF-derived lung fibroblasts were conditioned in low-serum media for 24 hours, as described in Methods. 24 hours later, culture media was changed, and cells were incubated with FITC-dextran (70 kDa, 0.5 mg/mL) for 1 hour. Then, cells were washed 3 times with PBS. Macropinocytosis was measured by fluorescent microscopy (**A**) (scale bar: 25 μm) and flow cytometry (**B**) to measure fluorescence intensity of FITC-dextran in cells (*n* = 5 for **A** and *n* = 8 for **B** in each condition). (**C** and **D**) Cells were treated with vehicle (Veh) or EIPA (12.5 μM) for 24 hours. Then, cells were harvested and subjected to Western blot (**C**) and qRT-PCR (**D**) to measure collagen 1 and α-SMA expression, as described in Supplemental Methods and Supplemental Table 1 (*n* = 5 each condition). (**E** and **F**) Control HLFs were treated with EIPA (12.5 μM) with or without TGF-β1 (10 ng/mL) for 24 hours. Then, cells were harvested and subjected to Western blot (**E**) and qRT-PCR (**F**), as described in Methods (*n* = 4 each condition). (**G**) Control HLFs were mixed with collagen 1 solution, as described in Methods, and treated with EIPA (12.5 μM) with or without TGF-β1 (10 ng/mL). Gel size was measured at 0 and 24 hours after collagen gelation (*n* = 4 for each condition). Data are shown as the mean ± SEM. *P < 0.05, vs. unstimulated or Control + Veh; ^†^*P* < 0.05, vs. IPF + Veh or TGF-β1 alone; significant comparisons by Student’s *t* test (**A** and **B**) or 1-way ANOVA (**C**–**G**).

Lung fibroblasts derived from IPF lung tissue possess a highcapacity for myofibroblast differentiation, ECM production, and resistance to cell death and cell invasion (12–14). We investigated whether macropinocytosis levels are elevated in IPF-derived lung fibroblasts compared with control fibroblasts. The uptake of fluorescent labeled large particles, such as 70 kDa dextran, is a widely accepted method to measure macropinocytosis in nonphagocytic cells (9, 15–17). Using fluorescence microscopy and flow cytometry techniques, IPF-derived lung fibroblasts showed increased levels of macropinocytosis compared with control cells, as measured by the uptake of 70kDa FITC-labeled dextran (Figure 1, A and B). Unlike other types of endocytosis, macropinocytosis is dependent on Na^+^/H^+^ proton exchangers (NHEs), also known as the solute carrier 9 family (SLC9s) (18–20). Thus, 5-(n-ethyl-n-isopropyl)-amiloride (EIPA), a known pan-SLC9 blocker, is considered as a first choice pharmacological inhibitor of macropinocytosis in biomedical research (16, 21–25). Consistent with prior reports, EIPA effectively inhibited macropinocytosis in IPF-derived lung fibroblasts, while neither affecting clathrin-dependent endocytosis nor expression of caveolin-1, a key protein in caveolin-mediated endocytosis (Supplemental Figure 1, A–C; supplemental material available online with this article; https://doi.org/10.1172/JCI197651DS1). We tested whether EIPA can inhibit profibrotic responses in IPF-derived lung fibroblasts. As shown in Figure 1, C and D, EIPA significantly downregulated collagen 1 (ECM protein) and α-SMA, a marker of myofibroblast transformation, protein and mRNA expression in IPF-derived lung fibroblasts. Similar results were found in TGF-β1–stimulated control lung fibroblasts, where EIPA attenuated collagen 1 production, myofibroblast transformation, and cell contractility (Figure 1, E–G). Of note, we observed a mild, but not statistically significant, decrease in fibronectin 1 (*FN1*) expression in response to EIPA in activated human lung fibroblasts (HLFs) (Supplemental Figure 1D). These data suggest that macropinocytosis activity is elevated in activated or IPF-derived lung fibroblasts and that inhibition of this process leads to decreased profibrotic responses in these cells.

### Macropinocytosis regulates profibrotic responses in activated HLFs by regulating AA-mediated mTORC1 activation.

**Figure 2:**
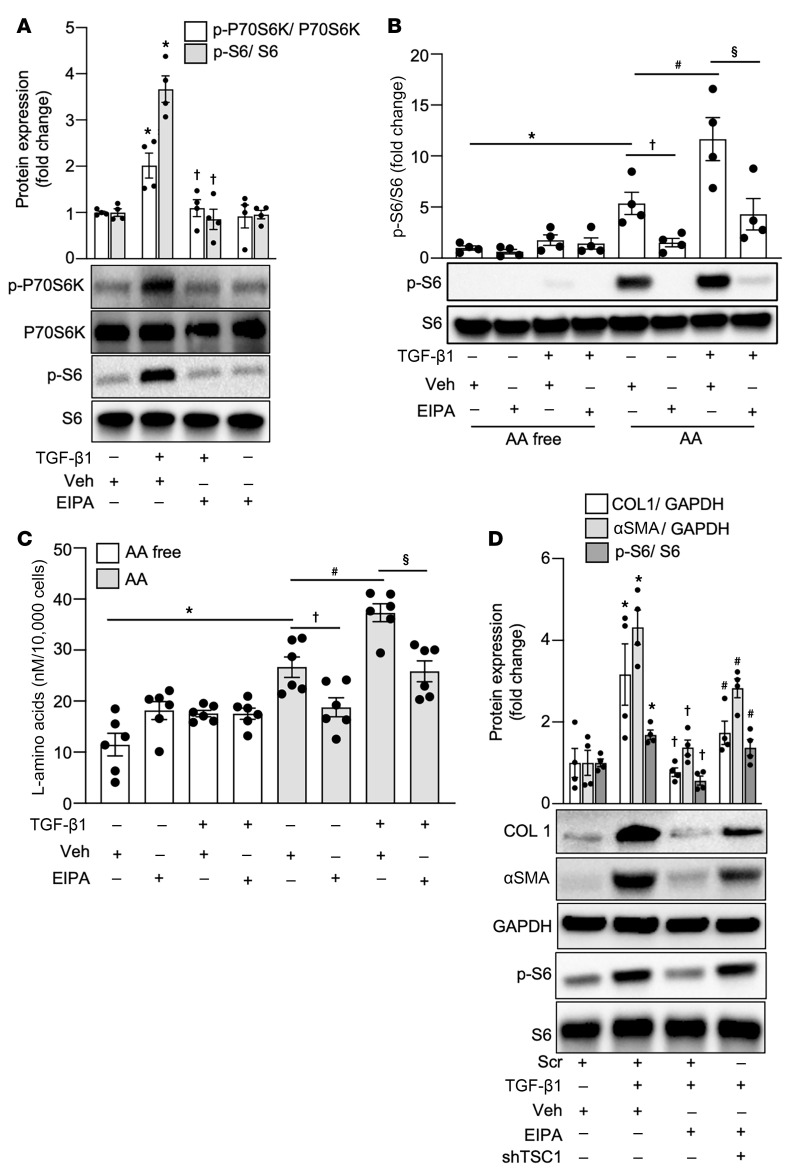
Macropinocytosis inhibition ameliorates mTORC1 signaling by regulating AA uptake in lung fibroblasts. (**A**) Control HLFs were treated with EIPA (12.5 μM) with or without TGF-β1 (10 ng/mL) for 24 hours. Then, p-P70S6K and p-S6 were measured by Western blot, as described in Methods (*n* = 4 each condition). (**B** and **C**) Cells were treated with EIPA (12.5 μM) with or without TGF-β1 (10 ng/mL) for 4 hours in AA-free and AA-supplemented conditions, as described in Methods. Then, cells were harvested to measure p-S6 by Western blot (**B**) and L-AA assay (**C**), as described in Methods (*n* = 4 or 6). (**D**) Cells were lentivirally transfected with scramble (Scr) or shTSC1, as described in Methods. Then, cells were treated with EIPA (12.5 μM) with or without TGF-β1 (10 ng/mL) for 24 hours, and cells were harvested, and COL1, α-SMA, and p-S6 were measured by Western blot (*n* = 4 each condition). Data are shown as the mean ± SEM. *P < 0.05, vs. Veh or Veh + AA free; ^†^*P* < 0.05, vs. TGF-β1 + Veh or Veh + AA; ^§^*P* < 0.05, vs. TGF-β1 + Veh + AA; ^#^*P* < 0.05, vs. Scr + TGF-β1 + EIPA; significant comparisons by 1-way ANOVA.

Previous reports suggest that macropinocytosis tightly regulates mTORC1 activation by regulating cytosolic AA levels, which can be obtained by direct uptake of free AAs or proteins (e.g., serum albumin) from the extracellular environment (9–11, 19). In turn, mTORC1 activation is one of the central signaling pathways in promoting profibrotic responses in fibroblasts by activating multiple downstream signaling pathways and regulating cell metabolism (26–28). Accordingly, we found that EIPA inhibits the phosphorylation of p70S6K and S6, which are representative of mTORC1 activation and metabolic reprogramming, in TGF-β1–activated lung fibroblasts in low-serum (1%) culture medium without affecting cell viability (Figure 2A and Supplemental Figure 2, A and B). We also observed that EIPA effectively downregulated extracellular acidification rate (Supplemental Figure 2C). Interestingly, withdrawal of AAs, using AA-free cell culture media, completely abrogated mTORC1 activation, even in the presence of TGF-β1 (Figure 2B). The addition of AAs resulted in the upregulation of phospho-S6 in lung fibroblasts, which was further enhanced by TGF-β1 treatment (Figure 2B). EIPA significantly attenuated S6 activation under AA-supplemented conditions (Figure 2B). Furthermore, we found that macropinocytosis regulates the amount of cytosolic AAs under these conditions, suggesting that under profibrotic conditions (e.g., under TGF-β1 stimulation), extracellular AAs play a significant role in mTORC1 activation and that macropinocytosis inhibition can significantly inhibit this process (Figure 2C). Indeed, we found that free AA or albumin supplementation increased *COL1A1* and *ACTA2* mRNA levels compared with AA-free conditioned cells, and this effect was inhibited by EIPA treatment (Supplemental Figure 3A). Finally, mTORC1 activation by TSC1 silencing, a negative regulator of mTOR, attenuated the antifibrotic effects of EIPA (Figure 2D). Whereas, shTSC1 alone significantly upregulated the expression of COL1 and aSMA at the protein and mRNA levels in TGF-b1 stimulated lung fibroblasts (Supplemental Figure 2F and Supplemental Figure 10, C and F). These data suggest that macropinocytosis regulates the uptake of extracellular AAs, which, in turn, leads to mTORC1 activation and increased profibrotic responses in lung fibroblasts.

### MEOX1 is regulated by macropinocytosis and mTORC1 in activated HLFs.

**Figure 3:**
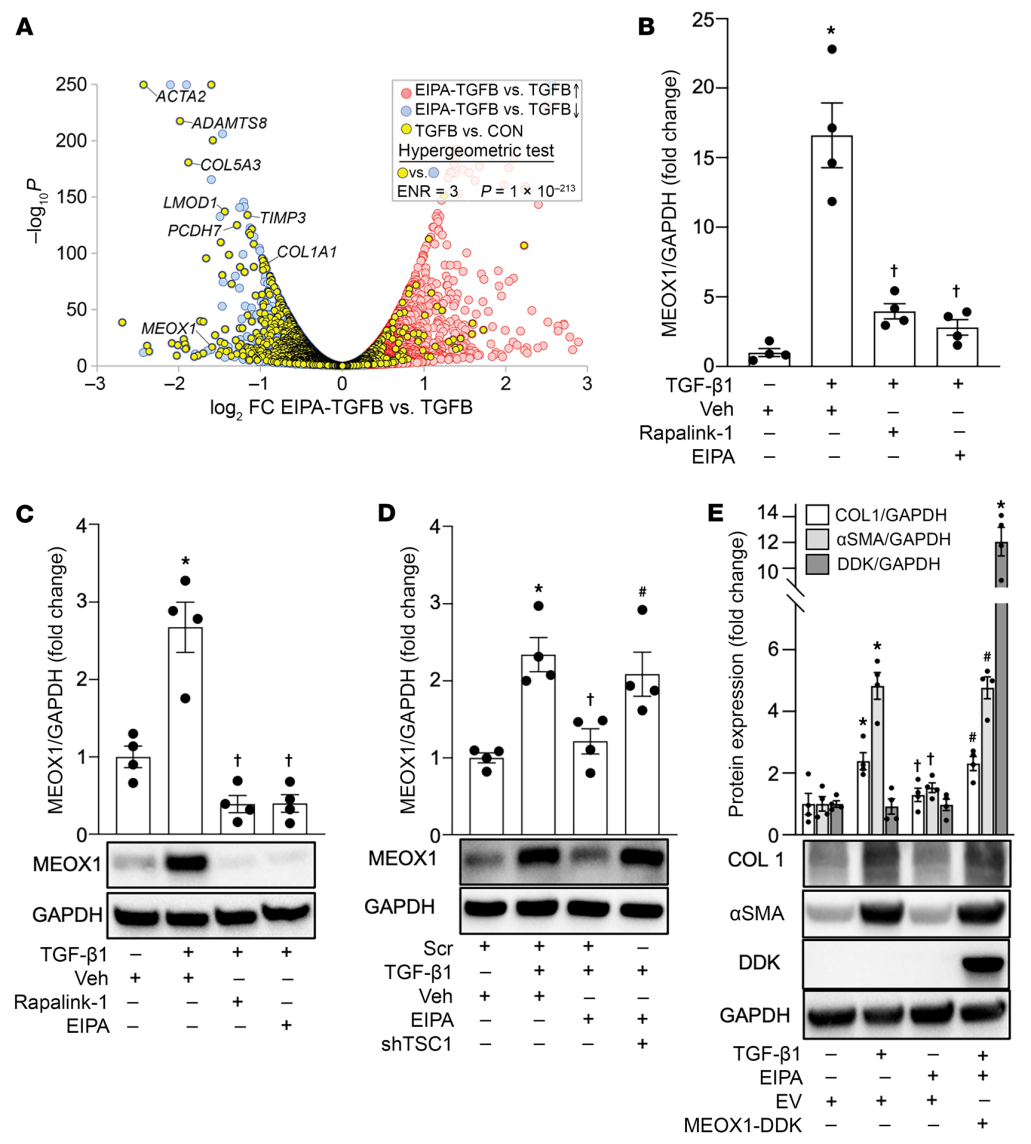
Macropinocytosis inhibition regulates MEOX1 expression in activated lung fibroblasts. (**A**) Volcano plot representing treatment of MRC5 HLFs with and without EIPA in the presence of TGFB. Overlaid in yellow is a set of TGFβ1-induced genes from a separate RNA sequencing experiment in MRC5 HLFs treated with TGF-β1 (10 ng/mL) or vehicle. MRC-5 cells were treated with EIPA (12.5 μM) with or without TGF-β1 (10 ng/mL) for 24 hours. Then, RNA was isolated and subjected to bulk RNA sequencing, as described in Methods (*n* = 3). (**B** and **C**) Control HLFs were treated with EIPA (12.5 μM) and Rapalink-1 (10 nM) with or without TGF-β1 (10 ng/mL) for 24 hours. Then, MEOX1 was measured at mRNA and protein levels by qRT-PCR (**B**) and Western blot (**C**), respectively (*n* = 4 each condition). (**D**) Control HLFs were lentivirally transfected with Scr or shTSC1, as described in Methods. Then, cells were treated with vehicle (Veh) or EIPA (12.5 μM) for 24 hours. Then, cells were harvested and subjected to Western blot to measure MEOX1 expression. (**E**) Control HLFs were lentivirally transfected with empty vector (EV) or pLenti-MEOX1-DDK, as described in Methods. Then, cells were treated with TGF-β1 (10 ng/mL) for 24 hours or not. Protein levels of COL1 and α-SMA were measured by Western blot, as described in Methods (*n* = 4 each condition). Data are shown as the mean ± SEM. *P < 0.05, vs. unstimulated or Scr alone; ^†^*P* < 0.05, vs. TGF-β1 alone; ^#^*P* < 0.05, TGF-β1+EIPA; significant comparisons by hypergeometric test (**A**) or 1-way ANOVA (**B**–**E**).

We next aimed to evaluate the effect of EIPA treatment on the TGF-β1–induced (TGFB-induced) transcriptional program in MRC5 HLFs. Consistent with a robust repressive effect of EIPA on the TGFB-induced transcriptional program, TGFB-induced genes were strongly enriched among EIPA-repressed genes (Figure 3A) (29). TGFB-induced genes repressed by EIPA treatment included several with well established roles in lung fibrosis such as *ACTA2* and *COL1A1*. Notably, mesenchyme homeobox1 (*MEOX1*), recently identified as a key transcription factor in fibroblast activation and cardiac fibrosis, was among the repressed genes (30). Accordingly, we next investigated whether MEOX1 was regulated by macropinocytosis-mediated mTORC1 activation and contributed to the profibrotic phenotype of activated HLFs. We found that both EIPA and Rapalink-1, a known mTORC1 inhibitor, downregulated MEOX1 mRNA and protein expression levels (Figure 3, B and C). However, the inhibitory effect of EIPA on MEOX1 expression was ameliorated in TSC1-deficient cells, suggesting that macropinocytosis inhibition downregulates the expression of MEOX1 by regulating mTORC1 in activated lung fibroblasts (Figure 3D).

Silencing of regulatory-associated protein of mTOR (RAPTOR), a key component of mTORC1, but not mTORC2, inhibited MEOX1 expression in TGF-β1–activated HLFs (Supplemental Figure 3B and Supplemental Figure 10, B and E). Of note, we also found that activation of mTOR by shTSC1 significantly increased MEOX1 gene expression (Supplemental Figure 2D and Supplemental Figure 10, A and D), whereas silencing of MEOX1 significantly inhibited COL1 and α-SMA expression in activated HLFs (Supplemental Figure 3C). Finally, we found that overexpression of MEOX1 reversed the antifibrotic effect of EIPA (Figure 3E) and that EIPA significantly ameliorated the binding of MEOX1 to *COL1A1* and *ACTA2* genes promoters (Supplemental Figure 3D) These data suggest that macropinocytosis inhibition negatively affects profibrotic responses in fibroblasts by regulating an mTORC1/MEOX1 signaling axis.

### Macropinocytosis inhibition attenuates lung fibrosis in Bleo-injured mice.

**Figure 4:**
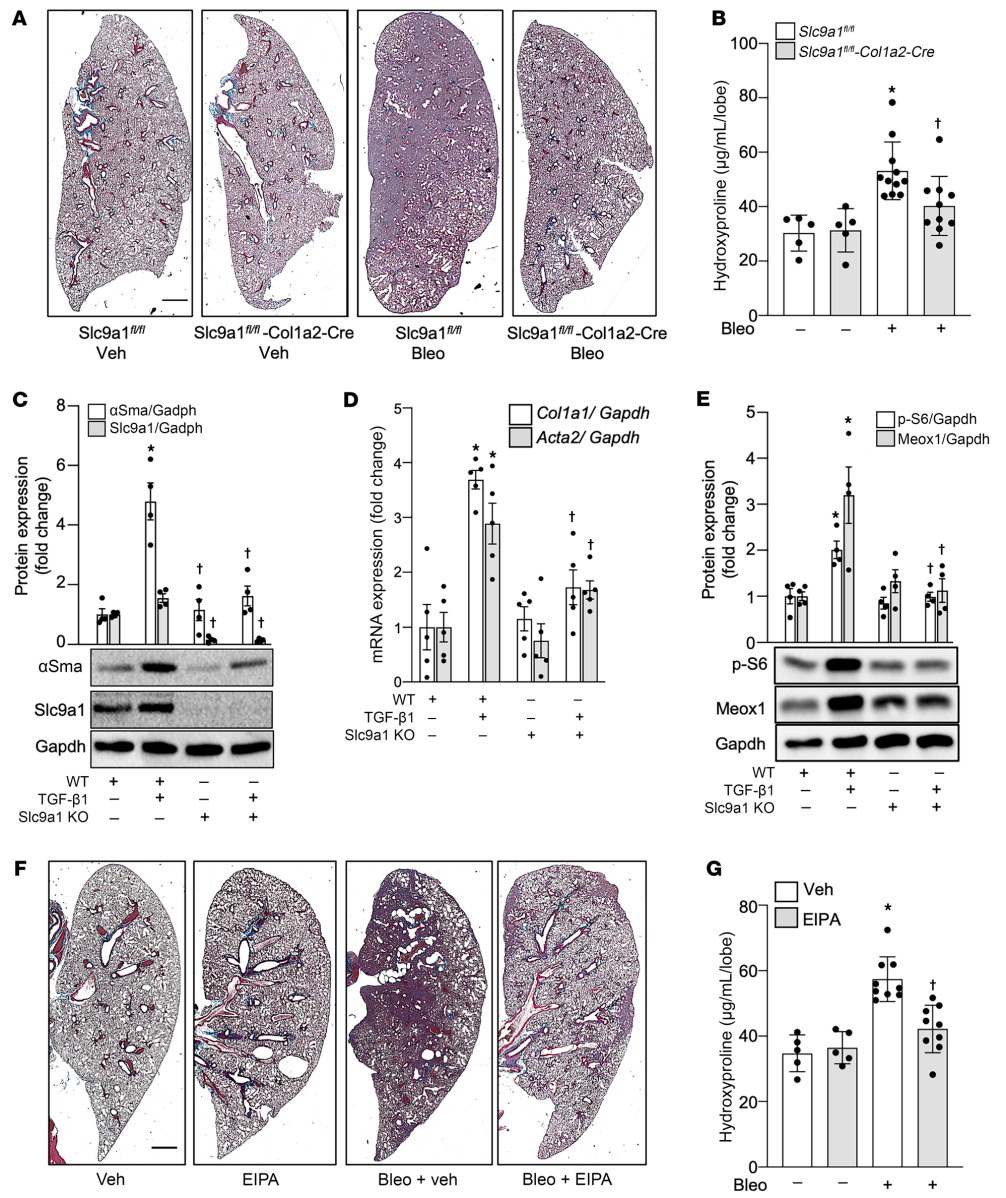
Macropinocytosis inhibition attenuates pulmonary fibrosis in Bleo-injured mice. (**A** and **B**) *Slc9a1^fl/fl^* (WT) or *Slc9a1^fl/fl^* Col1a2^Cre-ER(T)+/0^ (Slc9a1 fCKO) mice were treated with tamoxifen to induce Slc9a1 knockout in fibroblasts, as described in Methods. Then, mice were exposed to Bleo-induced pulmonary fibrosis, as described in Methods. At day 21 after Bleo exposure, lungs were harvested, stained with Masson’s trichrome staining (scale bar: 1,000 μm) (**A**) and subjected to hyproxyproline assay (**B**) (*n* = 5 for sham groups, *n* = 10–11 for Bleo groups). (**C**–**E**) Mouse lung fibroblasts from WT and Slc9a1 fCKO mice were isolated, as described in Methods. Cells were stimulated with TGF-β1 (10 ng/mL) for 24 hours. Then, cells were subjected to Western blot or qRT-PCR, as described in Methods. (**F** and **G**) WT mice were exposed to Bleo to induce pulmonary fibrosis, as described in Methods. 10 days after Bleo exposure, mice were intraperitoneally treated with EIPA (10 mg/kg) or vehicle (Veh) every other day until the end of experiment. At day 21 after Bleo exposure, lungs were harvested, stained with Masson’s trichrome staining (*n* = 3–4) (scale bar: 1,000 μm). (**F**) and subjected to hyproxyproline assay (**G**) (*n* = 5 for sham groups, *n* = 9 for Bleo groups). Data are shown as the mean ± SEM. *P < 0.05, vs. No Bleo (sham) or unstimulated; ^†^*P* < 0.05, vs. Bleo or TGF-β1 alone; significant comparisons by 1-way ANOVA (**B**–**E** and **G**).

Next, we examined whether genetic silencing or pharmacologic inhibition of macropinocytosis can ameliorate pulmonary fibrosis in bleomycin-injured (Bleo-injured) mice. Previously, Nhe1/Slc9a1 deficiency was shown to markedly inhibit macropinocytosis in vitro and in vivo (31). Indeed, we found that Slc9a1-deficient lung fibroblasts exhibited decreased macropinocytosis activity compared with their WT counterparts, without affecting clathrin-dependent endocytosis or caveolin1 expression (Supplemental Figure 4). We found that deficiency of Slc9a1 in fibroblasts significantly attenuated pulmonary fibrosis in mice subjected to intratracheal Bleo administration (Figure 4, A and B, and Supplemental Figure 5A). Furthermore, Slc9a1 deficiency in mouse lung fibroblasts led to a decrease in Col1a1 and α-SMA expression, mTORC1 activation, and Meox1 after TGF-β1 stimulation (Figure 4, C–E). Consistently, delayed administration of a macropinocytosis inhibitor (EIPA, 10 mg/kg) significantly reduced macropinocytosis and lung fibrosis, as determined by Trichrome and H&E staining and hydroxyproline assay, without affecting lung inflammation in Bleo-injured mice (Figure 4, F and G, Supplemental Figure 5B, and Supplemental Figure 6). Accordingly, total lung homogenates showed a significant downregulation of profibrotic gene expression and MEOX1 expression in fibroblast-specific Slc9A1 deficient and EIPA-treated mice (Supplemental Figure 7). Taken together, our data strongly suggest that macropinocytosis inhibition ameliorates pulmonary fibrosis in mice.

### Macropinocytosis inhibition regulates the expression of profibrotic genes in different fibroblast subpopulations in IPF-derived PCLSs.

**Figure 5:**
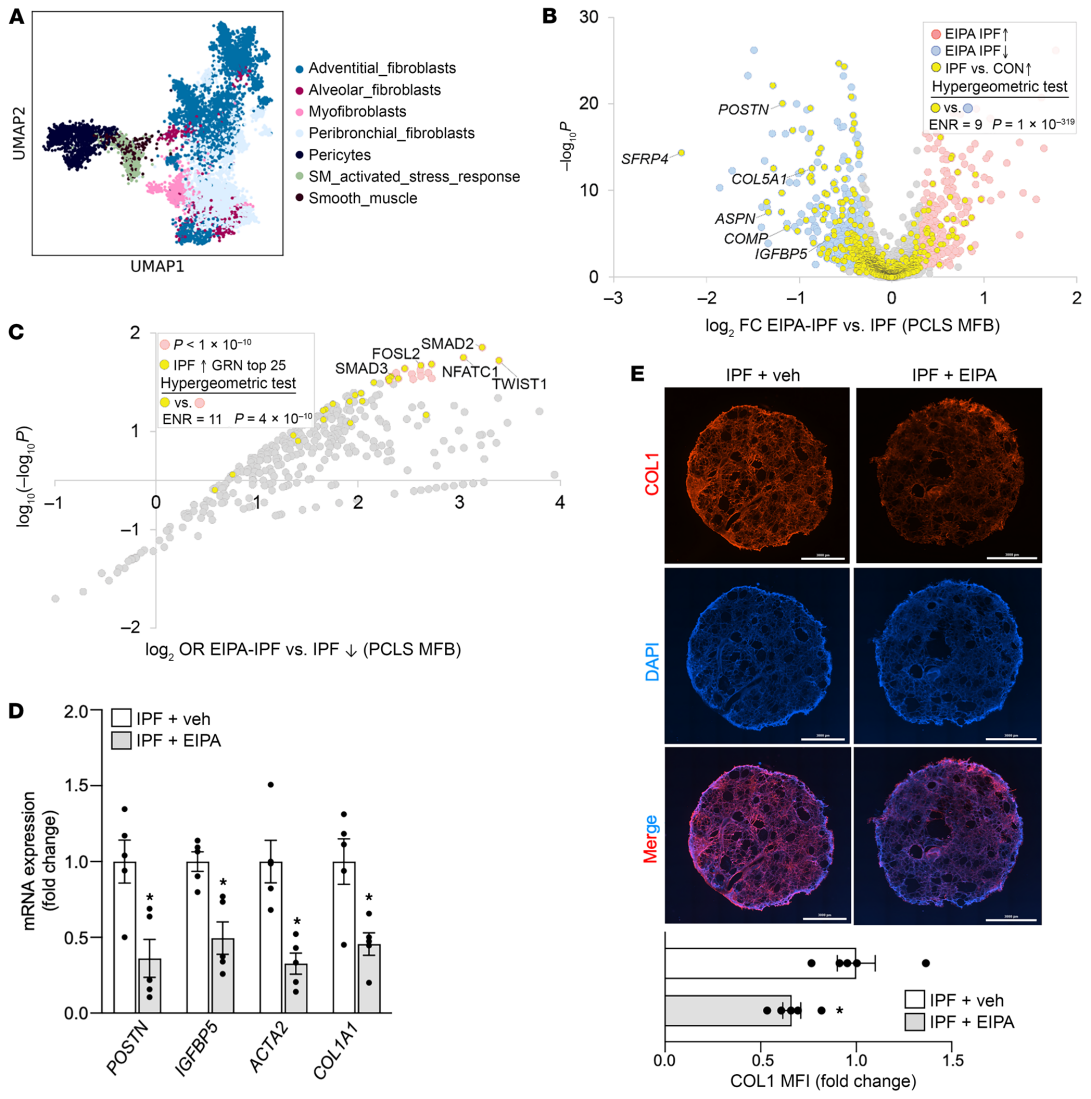
EIPA inhibits profibrotic responses in IPF-derived PCLSs. IPF-derived PCLSs were treated with EIPA (12.5 μM), as described in Methods. (**A**) UMAP plot represents different mesenchymal cell populations identified in IPF PCLSs (*n* of 4 for DMSO and *n* of 3 for EIPA-treated PCLSs). (**B**) Volcano plot representing treatment of IPF PCLSs with and without EIPA, as described in **A**. Overlaid in yellow is a set of myofibroblast IPF-induced genes from a separate PCLS experiment comparing IPF + DMSO (*n* of 4) and control (n of 4) PCLS samples, which were reused from McKenna et al. (29). (**C**) Regulatory network plot for genes repressed in EIPA-treated IPF PCLSs compared with IPF alone. TFs with the largest and most significant footprints within the gene set are distributed toward the top right of the plot. (**D**) After treatment, PCLSs were lysed and subjected to total RNA isolation. mRNA levels of POSTN, IGFBP5, ACTA2, and COL1A1 were measured with qRT-PCR, as described in Methods (*n* = 5 for each condition). (**E**) After treatment, PCLSs were washed with PBS and fixed for COL1 staining, as described in Methods. The COL1 fluorescent intensity was measured with ImageJ software (NIH) (*n* = 5 for each condition). Scale bar: 3,000 μm. Data are shown as the mean ± SEM. *P < 0.05, vs. No Bleo (sham); ^†^*P* < 0.05, vs. Bleo alone; significant comparisons by hypergeometric test (**B** and **C**) and Student’s *t* test (**D** and **E**).

Pulmonary fibroblasts represent a heterogenous cell population (32). To determine the effect of macropinocytosis inhibition on different fibroblast subpopulations in IPF lung, we treated precision cut lung slices (PCLSs) derived from individuals with IPF with EIPA and compared them with vehicle-treated PCLS from the same individuals. Single-cell RNA sequencing identified 7 cell populations of mesenchyme origin (Figure 5A and Supplemental Figure 8). Consistent with the results obtained from isolated fibroblasts and Bleo-injured mice, we observed a potent repressive effect of EIPA on the IPF-induced profibrotic gene expression in myofibroblasts (Figure 5B), known cells found to contribute to the progression of pulmonary fibrosis in mice and patients with IPF (33). Additionally, EIPA exerted a measurable effect on the other 6 mesenchymal subpopulations (Supplemental Figure 8). Gene regulatory network (GRN) analysis predicts functional footprints for transcription factors within clinical gene sets of interest (34, 35). To identify candidate transcription factors (TFs) affected by EIPA in IPF, we carried out GRN analysis on genes repressed by EIPA treatment of IPF PCLS myofibroblasts (Figure 5C) (29). Reiterating the strong antifibrotic effect of EIPA observed at the transcriptional level, the top ranked TFs from an IPF-induced PCLS myofibroblast GRN were strongly enriched among the EIPA-repressed PCLS myofibroblast GRN. Particularly strong footprints were observed for numerous TFs with established roles in pulmonary fibrosis, including NFATC1, TWIST1, and members of the SMAD family (Figure 5C), suggesting that EIPA may exert its antifibrotic effects by targeting one or more of these factors. We validated EIPA-mediated downregulation of profibrotic genes, such as periostin (POSTN), insulin growth factor binding protein-5 (IGFBP5), COL1A1, and ACTA2 by RT-qPCR in PCLS homogenates (Figure 5D). Finally, collagen 1 expression was also downregulated in EIPA-treated PCLSs compared with vehicle-treated PCLSs (Figure 5E).

### Imi inhibits fibroblast activation and pulmonary fibrosis.

**Figure 6:**
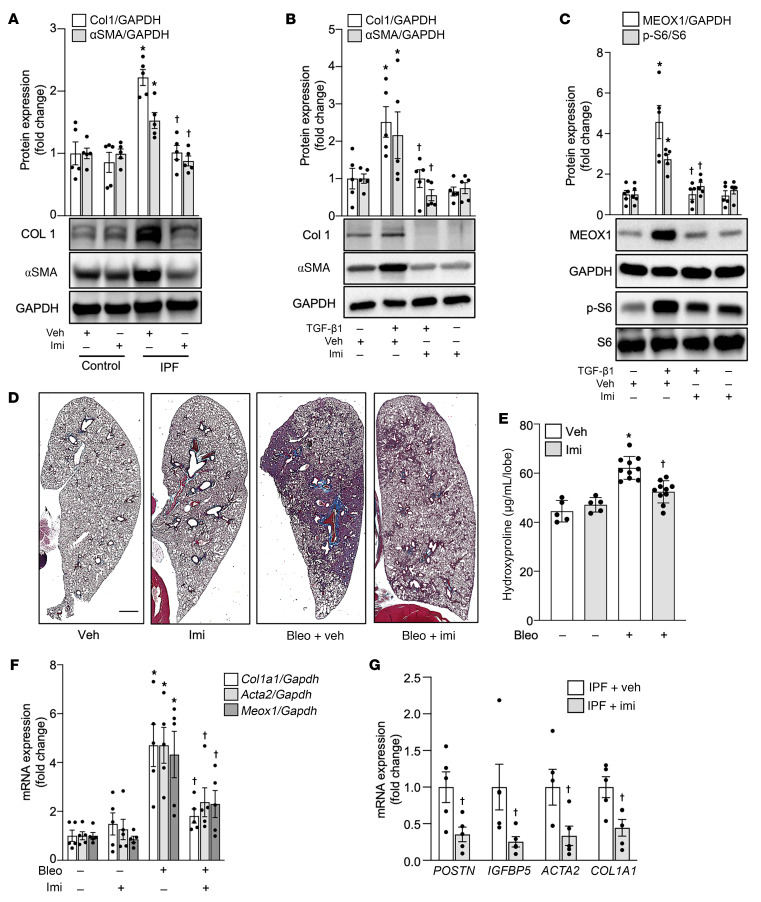
Imipramine ameliorates profibrotic responses in lung fibroblasts, Bleo-injured mice, and PCLSs. (**A**) Control and IPF-derived lung fibroblasts were treated with imipramine (Imi, 10 μM). 24 hours later cells were harvested, and COL1 and α-SMA were measured with Western blot (*n* = 4 each condition). (**B** and **C**) Control HLFs were treated with Imi (10 μM) with or without TGF-β1 (10 ng/mL) for 24 hours. Then, cells were harvested and subjected to Western blot to measure COL1, α-SMA, p-S6, and MEOX1 (*n* = 4 for each condition). (**D**–**F**) WT mice were exposed to Bleo-induced pulmonary fibrosis, as described in Methods. 10 days after Bleo exposure, mice were intraperitoneally treated with Imi (10 mg/kg) or vehicle (Veh) every other day until the end of the experiment. At day 21 after Bleo exposure, lungs were harvested, stained with Masson’s trichrome staining (*n* = 3–4) (**D**), and subjected to hyproxyproline assay (*n* = 5 or 10) (**E**) or mRNA assessment (*n* = 5) (**F**). (**G**) IPF-derived PCLSs were treated with Imi (10 μM), as described in Methods. Then, cells were lysed and mRNA levels of POSTN, IGFBP5, COL1A1, and ACTA2 were measured by qRT-PCR (*n* = 5 each condition). *P < 0.05, vs. unstimulated or Control or No Bleo (sham); ^†^*P* < 0.05, vs. TGF-β1 or IPF alone or Bleo alone; significant comparisons by 1-way ANOVA (**A**–**C**, **E**, and **F**) and Student’s *t* test (**G**).

A previous report suggested that Imi selectively inhibits macropinocytosis over other types of endocytosis in macrophages (21). We also found that Imi strongly inhibited macropinocytosis in IPF-derived lung fibroblasts without affecting clathrin-dependent endocytosis or caveolin-1 expression (Supplemental Figure 1A). Imi effectively inhibited profibrotic responses in IPF-derived and TGF-β1–induced lung fibroblasts as well as mTORC1/MEOX1 signaling (Figure 6, A–C, and Supplemental Figure 7C). Similar to EIPA, Imi significantly ameliorated pulmonary fibrosis in Bleo-injured mice and profibrotic responses in IPF-derived PCLSs (Figure 6, D–G, and Supplemental Figure 5C). Together, these results suggest that antifibrotic effects in IPF can be achieved by targeting macropinocytosis either with EIPA or through the potential repurposing of the FDA-approved drug Imi.

## Discussion

The observation that fibroblasts may undergo membrane ruffling followed by endocytosis has been documented as early as the mid-1980s (36). Furthermore, these studies have suggested that macropinocytosis in fibroblasts can be activated by either genetic intervention (e.g., Ras and Src pathway activation) or by growth factors such as platelet-derived growth factor (36–38). More recently, Zhang et al. demonstrated that TGF-β1, a potent profibrotic growth factor, induces macropinocytosis in pancreatic ductal adenocarcinoma cells (39). Indeed, we also observed a mild increase in macropinocytosis after TGF-β1 stimulation at early time points in primary HLFs (Supplemental Figure 1E), which were confirmed by the presence of mesenchymal and the absence of immune, epithelial, and endothelial markers (Supplemental Figure 11). Despite multiple reports suggesting that macropinocytosis occurs in fibroblasts and can be induced by various profibrotic stimuli such as growth factors or hypoxia, to the best of our knowledge, there are no reports describing the functional importance of this process in pulmonary fibrosis. Thus, we hypothesized that macropinocytosis may contribute to the profibrotic phenotype of activated fibroblasts and to the progression of pulmonary fibrosis. We found that macropinocytosis is upregulated in IPF-derived lung fibroblasts and that inhibition of this process ameliorates profibrotic responses in fibroblasts, Bleo-injured mice, and IPF-derived PCLSs (Figures 1, 4, and 5). Using single-cell transcriptomics, we found that EIPA exerts potent antifibrotic effects in different fibroblast subpopulations, including myofibroblasts, a known effector cell lineage in pulmonary fibrosis (40). Previously, it has been shown that collagen triple helix repeat containing 1–expressing (CTHRC1-expressing) fibroblasts play a critical role in pulmonary fibrosis (32). We found that CTHRC1 expression was highest in myofibroblasts compared with other fibroblast populations (Supplemental Figure 8G). We also identified fibroblast subpopulations expressing elevated levels of CTHRC1 (CTHRC1^hi^) and demonstrated robust inhibition of profibrotic transcriptional programs in these subpopulations by EIPA (Supplemental Figure 9).

Mechanistically, macropinocytosis is not a fully defined process. However, several reports have suggested that macropinocytosis is dependent on sodium-hydrogen exchangers, such as SLC9A1, which are known to induce intracellular pH increase (alkalinization) by exporting hydrogen protons into the extracellular environment, which, in turn, causes actin rearrangements and macropinocytosis (18, 41). Indeed, Slc9a1-deficient fibroblasts showed decreased macropinocytosis and an ameliorated profibrotic phenotype. Furthermore, Slc9a1 fibroblast conditional knockout mice were protected from Bleo-induced pulmonary fibrosis (Figure 4).

Interestingly, activation of SLC9A1 may, at the same time, decrease extracellular pH levels by releasing hydrogen protons into the extracellular milieu (42). Indeed, we found that extracellular acidification rate levels were elevated in our Mito Stress Assay (Agilent Technologies, Catalog 103015-100) after TGF-β1 stimulation and were downregulated by EIPA in lung fibroblasts (Supplemental Figure 2C). Given that the acidic environment has been shown to contribute to the myofibroblast transformation by regulating TGF receptor signaling, future studies can be conducted to study the possible link between SLC9A1-mediated profibrotic effects and changes in extracellular pH (43). Another potential area of investigation is to explore the role of syndecan-1 (SDC1) in macropinocytosis regulation in fibroblasts. It has been previously demonstrated that SDC1 promotes both pulmonary fibrosis and macropinocytosis (44, 45).

Macropinocytosis activation facilitates the uptake of protein and free AAs, which are directly sensed by mTORC1. This, in turn, activates signaling pathways that regulate vital intracellular processes, including protein and lipid synthesis, mitochondrial biogenesis, and glycolysis (9, 10, 46, 47). We found that macropinocytosis inhibition ameliorates mTORC1 signaling by regulating AA uptake in activated lung fibroblasts (Figure 2). mTORC1 has also been shown to contribute to the progression of fibroblast activation and pulmonary fibrosis (26, 27, 48). However, these preclinical and experimental findings were challenged when rapamycin, a nonspecific mTOR inhibitor, demonstrated limited success as a treatment for IPF compared with placebo in a short-term clinical study (49). Therefore, a precise understanding of mTORC1-mediated profibrotic signaling pathways will be required to develop either more specific mTORC1 inhibitors or those that target profibrotic signaling proteins downstream of mTORC1 activation (50, 51). Recent advances in this area have resulted in the development of a third generation of mTORC1 inhibitors, termed bi-steric mTORC1 inhibitors, which are more specific and effectively regulate downstream signaling proteins, such as S6 kinase (S6K) and 4E-binding protein 1 (4EBP1), with minimal or no effect on mTORC2 (51, 52). These inhibitors have already demonstrated therapeutic effects in experimental models of cancer and lymphangioleiomyomatosis (51–53). The potential antifibrotic activity of bi-steric mTORC1 inhibitors should also be tested in lung fibrosis models in the future.

MEOX1 belongs to a subfamily of antennapedia-like homeobox-containing transcription factors that recognize a palindromic DNA binding site sequence, TAATTA (54, 55). Early reports have demonstrated that MEOX1 plays critical roles in skeletal and mesenchymal tissue formation during embryogenesis, vascular cell activation, and senescence (55–57). Recent reports strongly suggest a pivotal role of MEOX1 in cardiac fibroblast activation and fibrosis (30, 58). More recently, a study conducted by Jin et al. has demonstrated that MEOX1 plays a key role in apoptosis resistance in myofibroblasts, a known profibrotic feature of IPF-derived lung fibroblasts (59). Consistent with previous findings, we also found that MEOX1 silencing ameliorated fibroblast activation (Supplemental Figure 3C). Furthermore, we found that MEOX1 expression and its binding to the COL1A1 and α-SMA promoter regions were downregulated by EIPA treatment (Figure 3C and Supplemental Figure 3D). RAPTOR silencing also inhibited MEOX1 expression, which suggests that the induction of MEOX1 expression in fibroblasts was mediated by macropinocytosis followed by mTORC1 activation (Supplemental Figure 3B).

Macropinocytosis is dysregulated in various health disorders, including cancer, and contributes to their pathobiology. Thus, the inhibition of this process may serve as a potential therapeutic strategy. To this end, an elegant study conducted by Lin et al. had screened 640 FDA-approved drugs and identified Imi, a tricyclic antidepressant, as a leading candidate to serve as a specific inhibitor of macropinocytosis by an unknown mechanism, without affecting phagocytosis or caveolin- or clathrin-mediated endocytosis (21). More recently, another tricyclic antidepressant, nortriptyline, has been found to potently inhibit macropinocytosis with prominent effects in blocking fatty acid uptake and repressing tumor growth (60). Since repurposing FDA-approved drugs can represent a more efficient and attractive strategy compared with de novo drug design, we tested the feasibility of Imi to regulate macropinocytosis and pulmonary fibrosis. As expected, we found that Imi downregulated macropinocytosis, similar to the effects of a known macropinocytosis inhibitor EIPA, without affecting clathrin-dependent endocytosis or caveolin-1 expression (Supplemental Figure 1). We also observed that Imi inhibits lung fibroblast activation and ameliorates fibrosis in mice and PCLS (Figure 6).

Taken together, our results strongly suggest that macropinocytosis inhibition is a feasible therapeutic strategy to ameliorate profibrotic responses in fibroblasts and pulmonary fibrosis. We further suggest that repurposing Imi, an FDA-approved antidepressant, as a macropinocytosis inhibitor can be considered for future clinical development as an antifibrotic therapy.

## Methods

Further information can be found in Supplemental Methods.

### Sex as a biological variable.

For human tissue-based studies, both male and female individuals were used. For mouse studies, we examined male and female animals, and similar findings are reported for both sexes.

### Statistics.

Data are expressed as mean ± SEM. Comparisons of mortality were made by analyzing Kaplan-Meier survival curves and log-rank tests to assess for differences in survival. For comparisons between 2 groups, we used Student’s unpaired 2-tailed *t* test. Statistical significance was defined as *P* < 0.05. One-way ANOVA, followed by Newman-Keuls or Tukey’s post test analysis, was used for analysis of more than 2 groups. The number of samples per group (*n*), or the number of experiments, is specified in the figure legends.

### Study approval.

This study was approved by the institutional review board at Baylor College of Medicine to use the deidentified patient specimens (protocol no. H-50814). All animal experimental protocols were approved by the Baylor College of Medicine Institutional Animal Care and Use Committee (protocol no. AN-8219).

### Data availability.

Data are available in the Supporting Data Values file or associated supplemental files. Bulk and single-cell RNA sequencing data were deposited in the Gene Expression Omnibus (GEO) database with accession numbers GSE298860 and GSE300087, respectively.

## Author contributions

IOR, CC, LJC, FP, NJM, and KT designed research studies. IOR, AKMS, SS, JDCG, ARWC, ERE, SAO, DK, CC, NJM, and KT conducted experiments and acquired and analyzed data. IOR, DCK, DS, and KT provided reagents. IOR, LJC, DS, FP, CC, NJM, and KT wrote the manuscript.

## Conflict of interest

The authors have declared that no conflict of interest exists.

## Funding support

This work is the result of NIH funding, in whole or in part, and is subject to the NIH Public Access Policy. Through acceptance of this federal funding, the NIH has been given a right to make the work publicly available in PubMed Central.

- National Institute of Arthritis, Musculoskeletal and Skin Diseases (AR074558).
- National Heart, Lung and Blood Institute (HL176934).
- Veterans Affairs Research Career Scientist award VA IK6 BX005647.

## Supplementary Material

Supplemental data

Supplemental data set 1

Unedited blot and gel images

Supporting data values

## Version 1 05/01/2026

In-Press Preview

## Version 2 06/15/2026

Electronic publication
