# Supplementary material for: Macropinocytosis inhibition attenuates profibrotic responses in lung fibroblasts and pulmonary fibrosis models
Source: J Clin Invest. 2026 Apr 30;136(12):e197651. doi: 10.1172/JCI197651 (PMC13262735; doi:10.1172/JCI197651)

Figure 1C

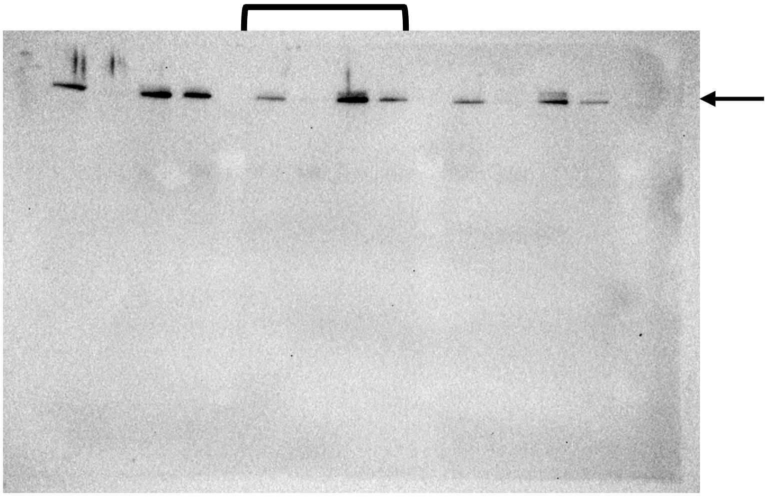

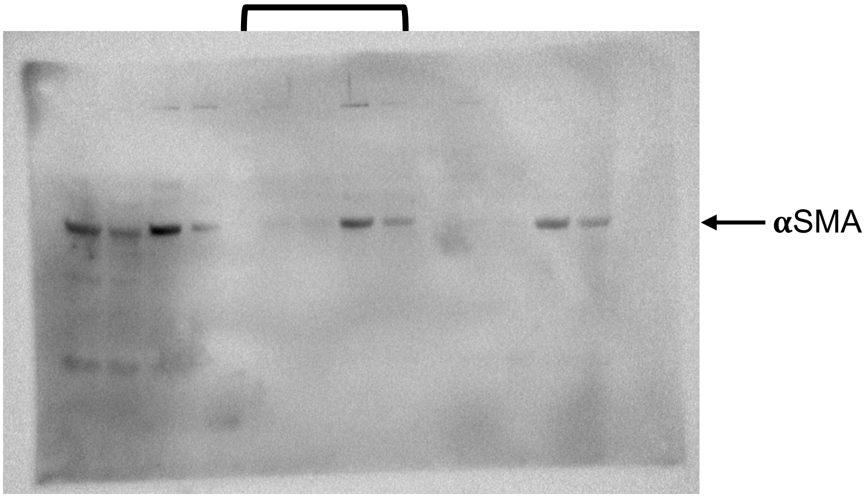

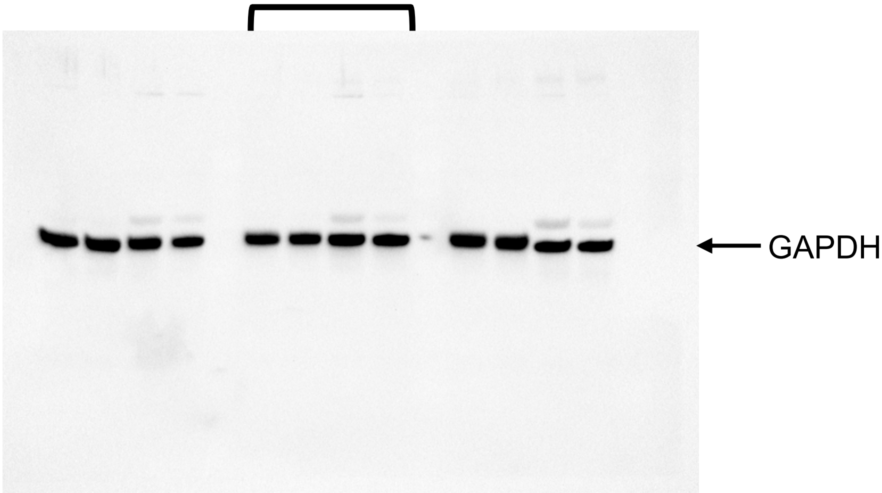

Figure 1E

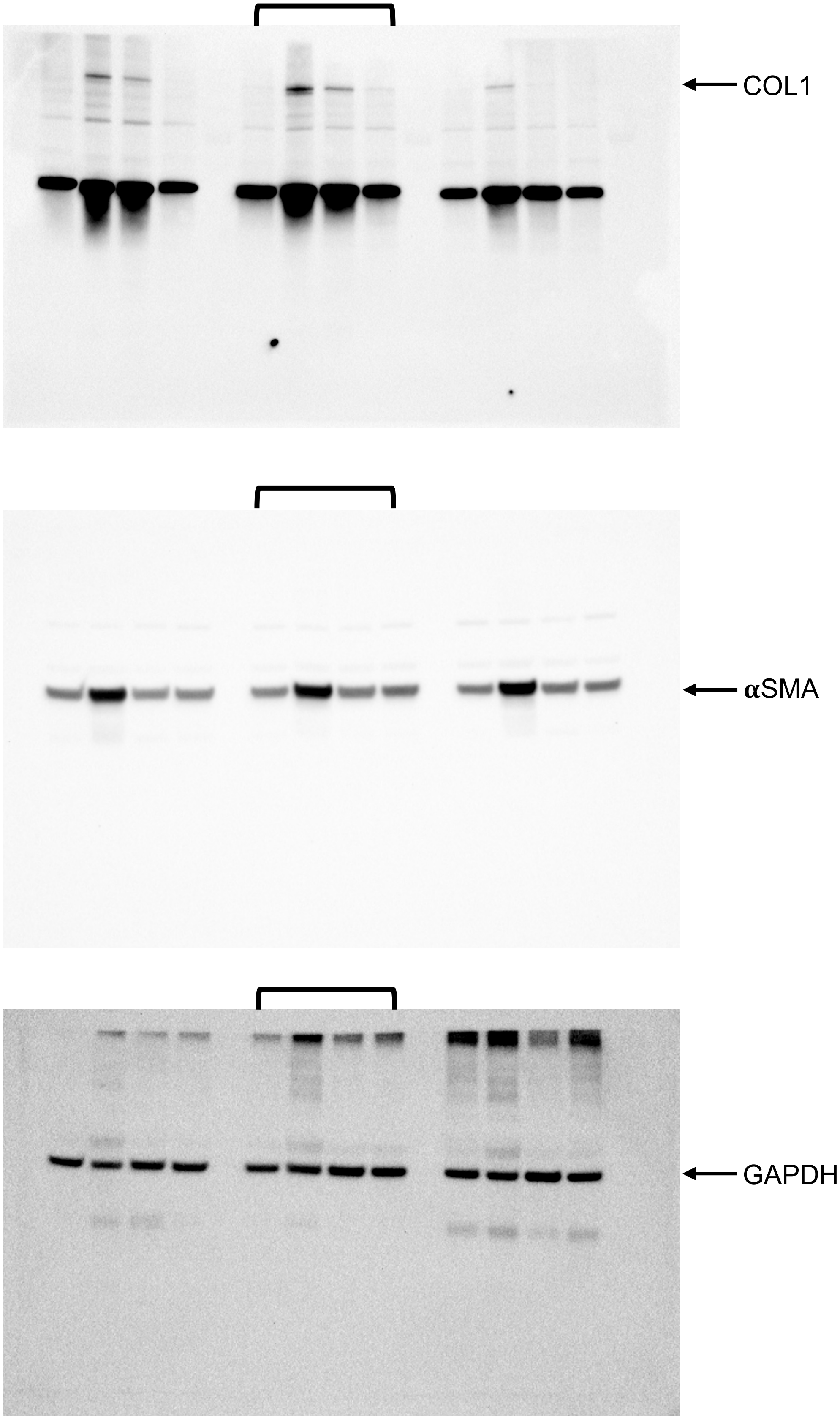

Figure 2A

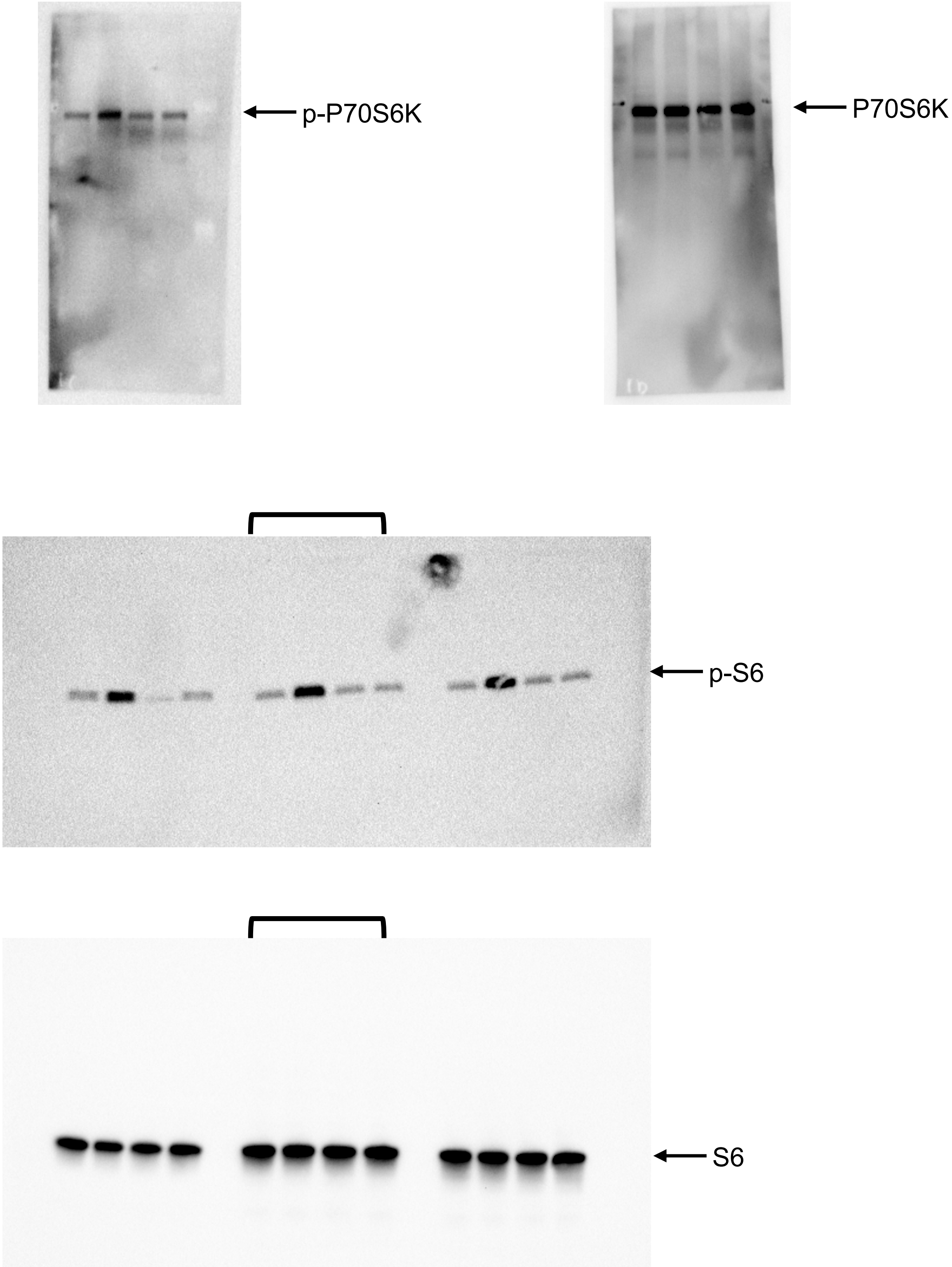

Figure 2B

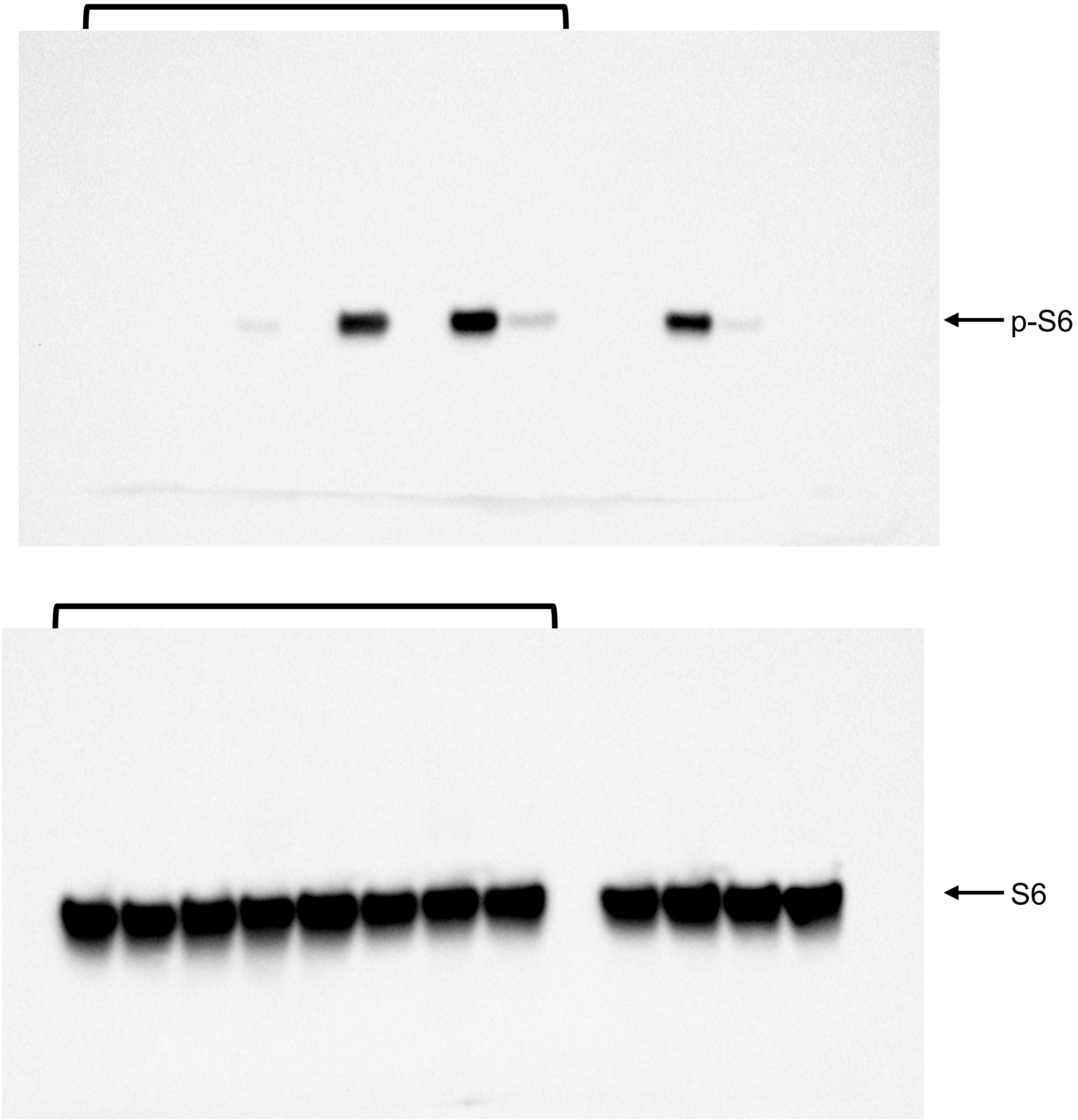

Figure 2D

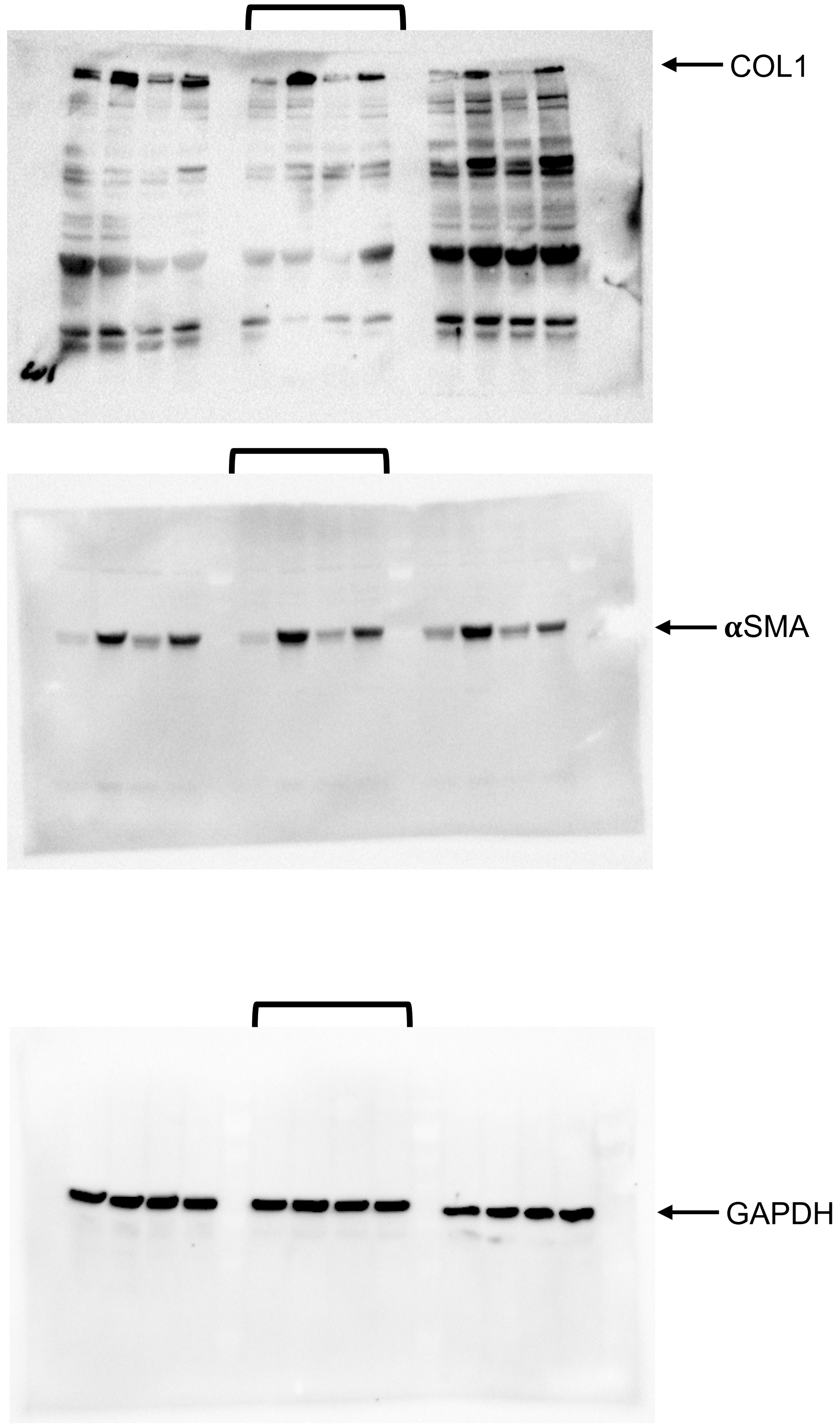

Figure 2D

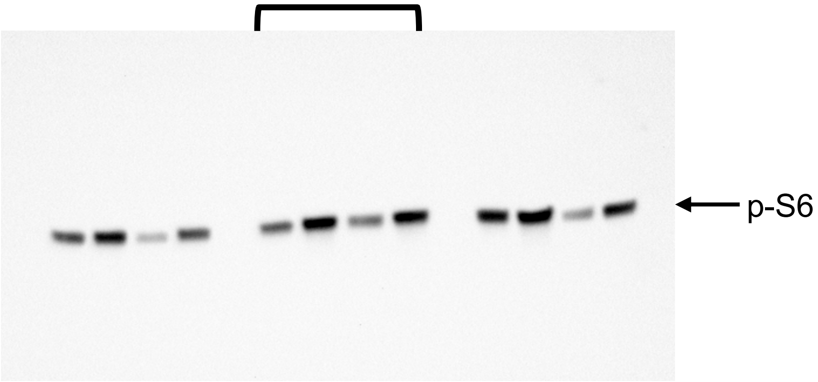

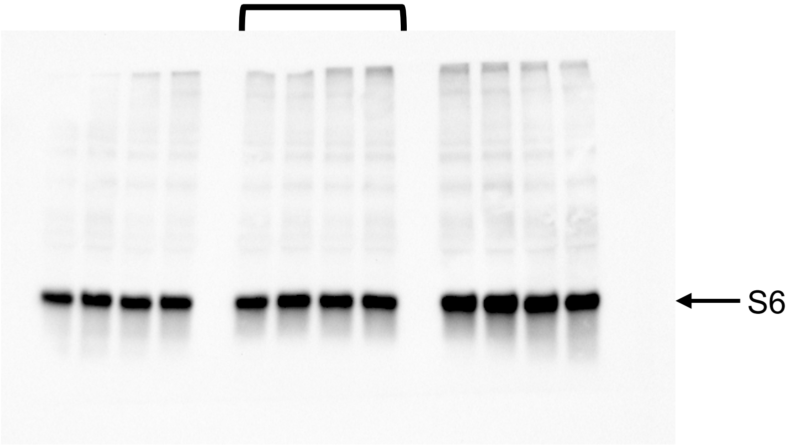

Figure 3C

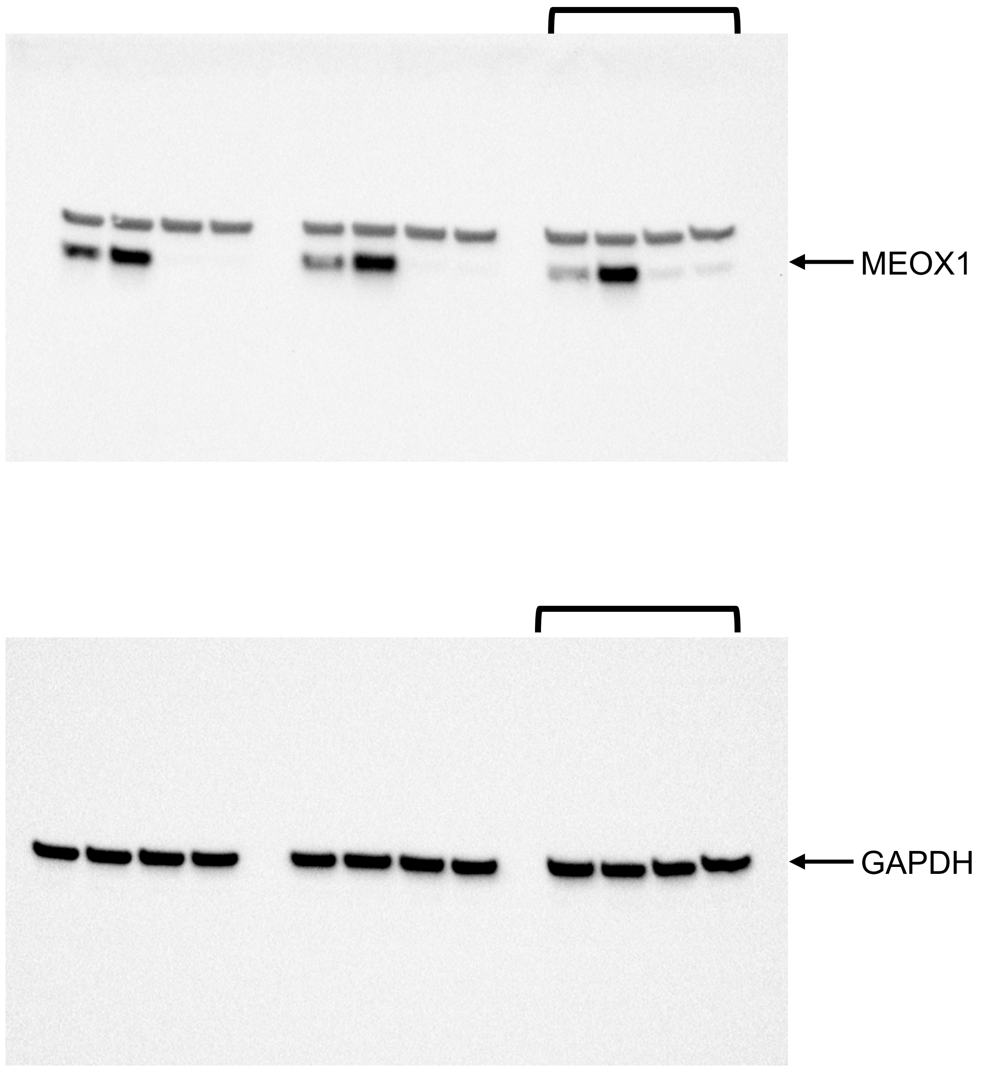

Figure3D

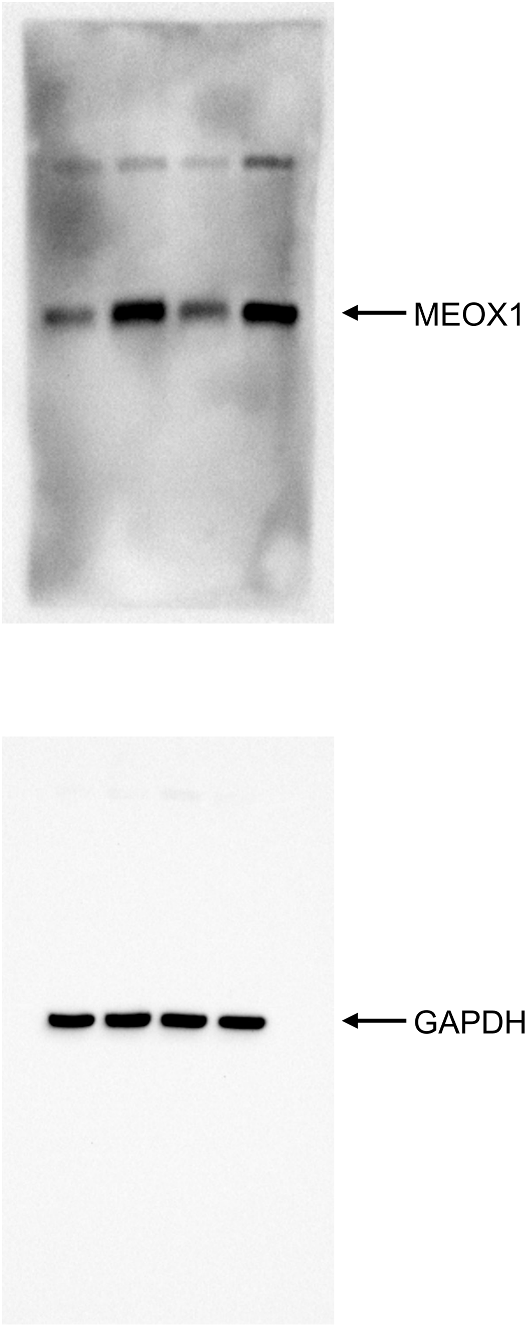

Figure3E

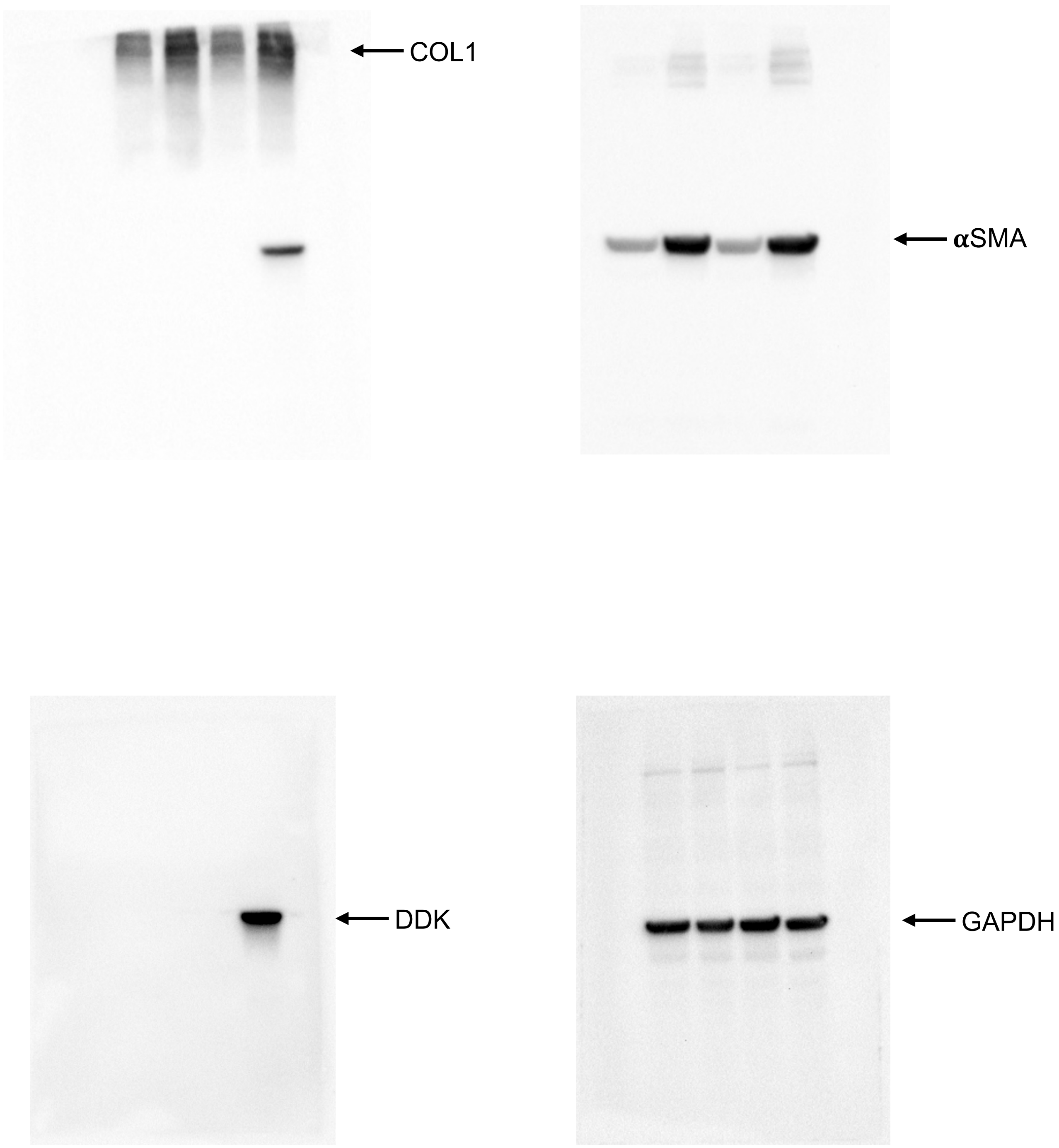

Figure 4C

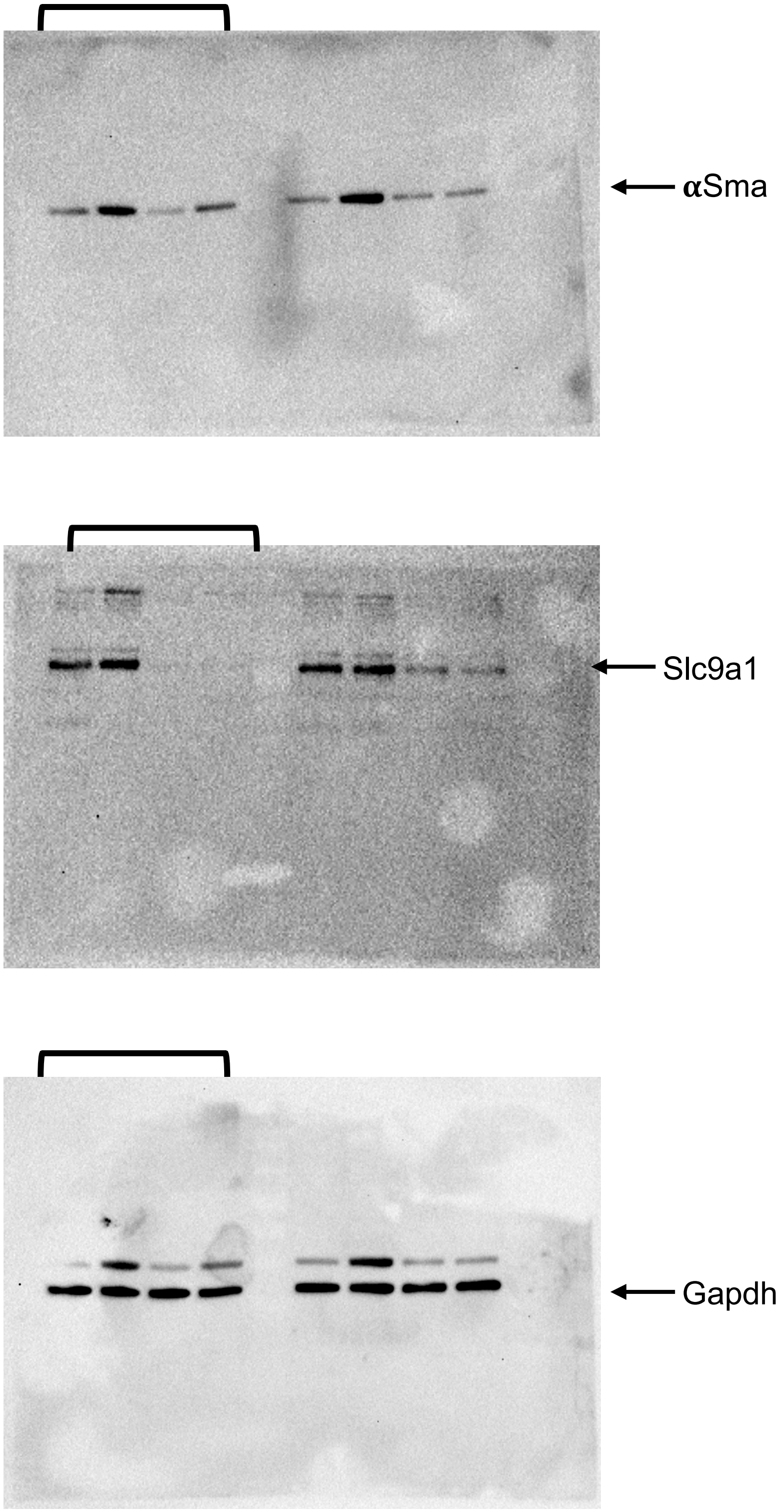

Figure 4E

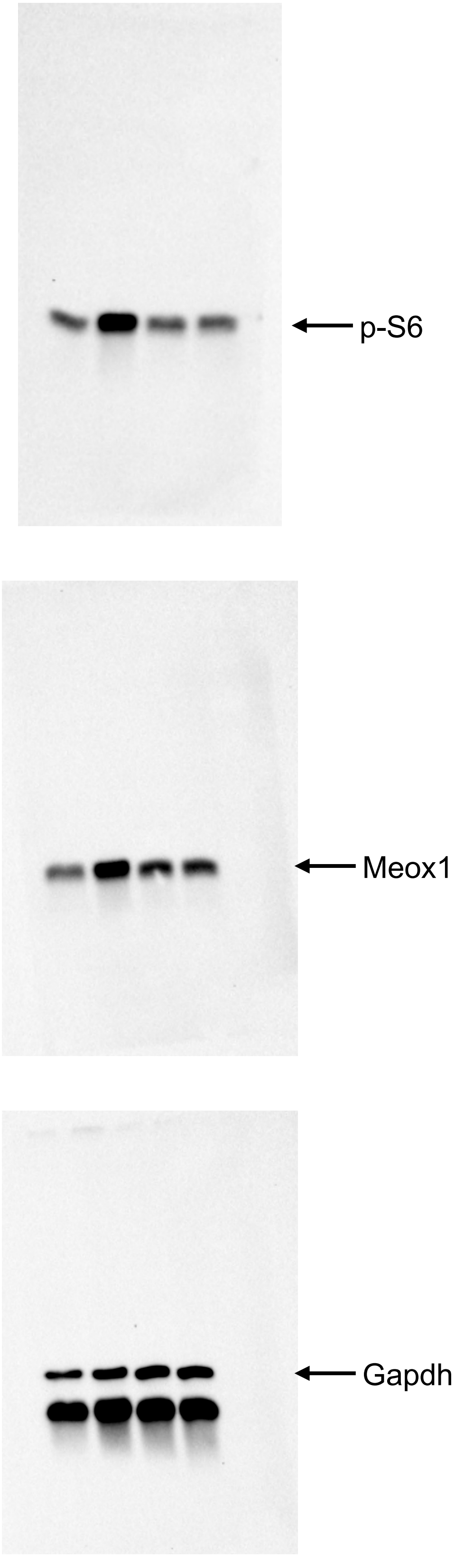

Figure 6A

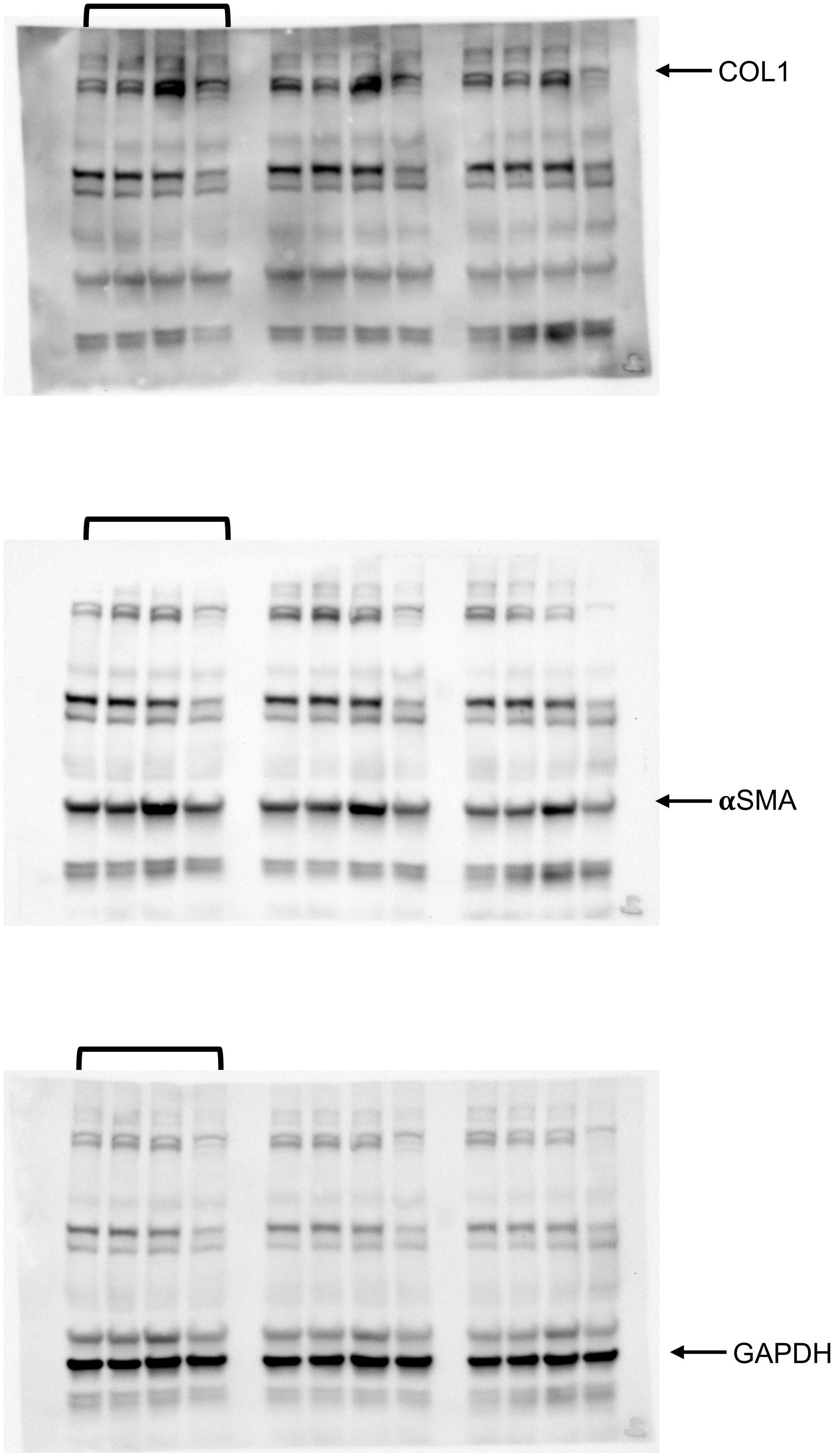

Figure 6B

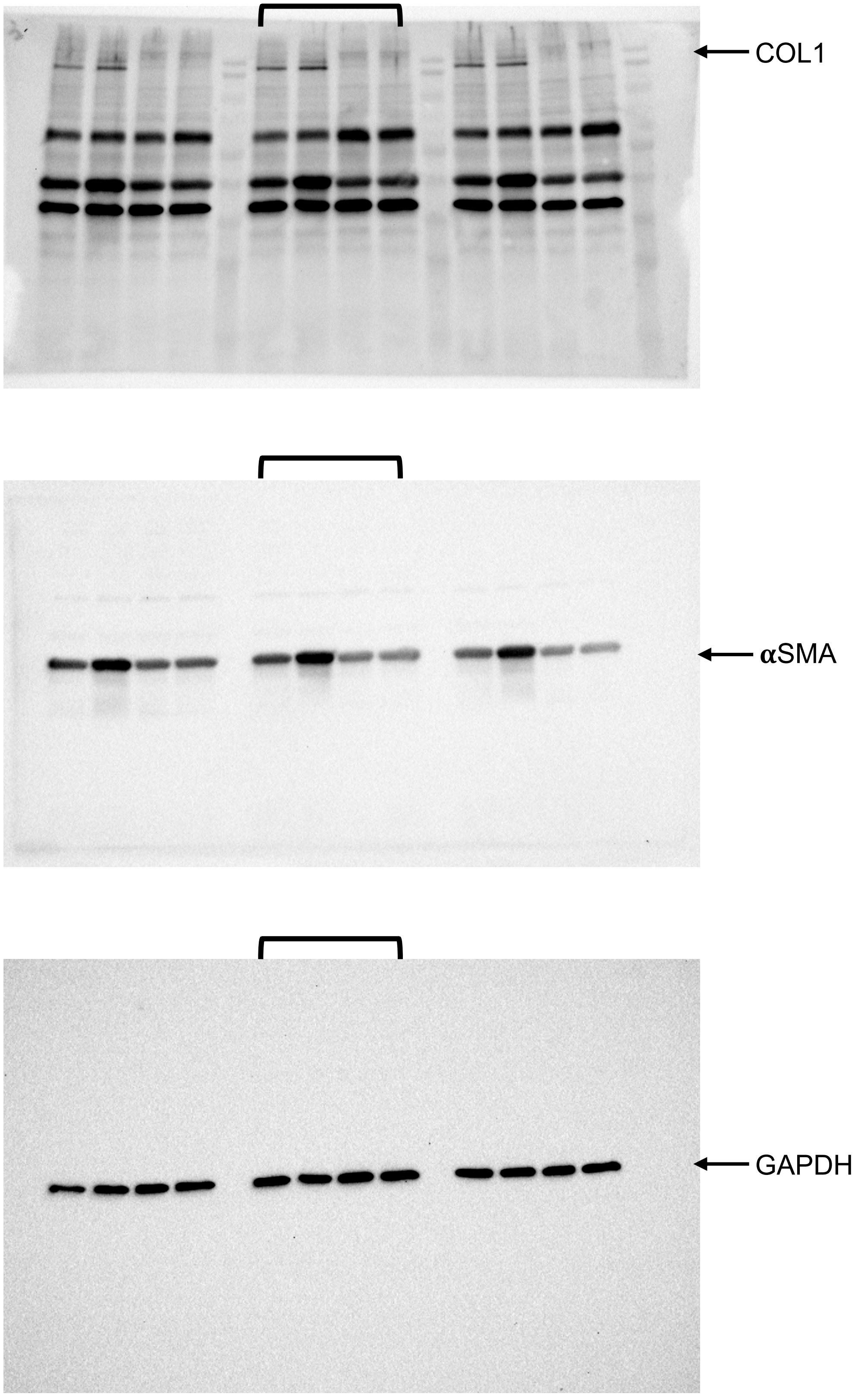

Figure 6C

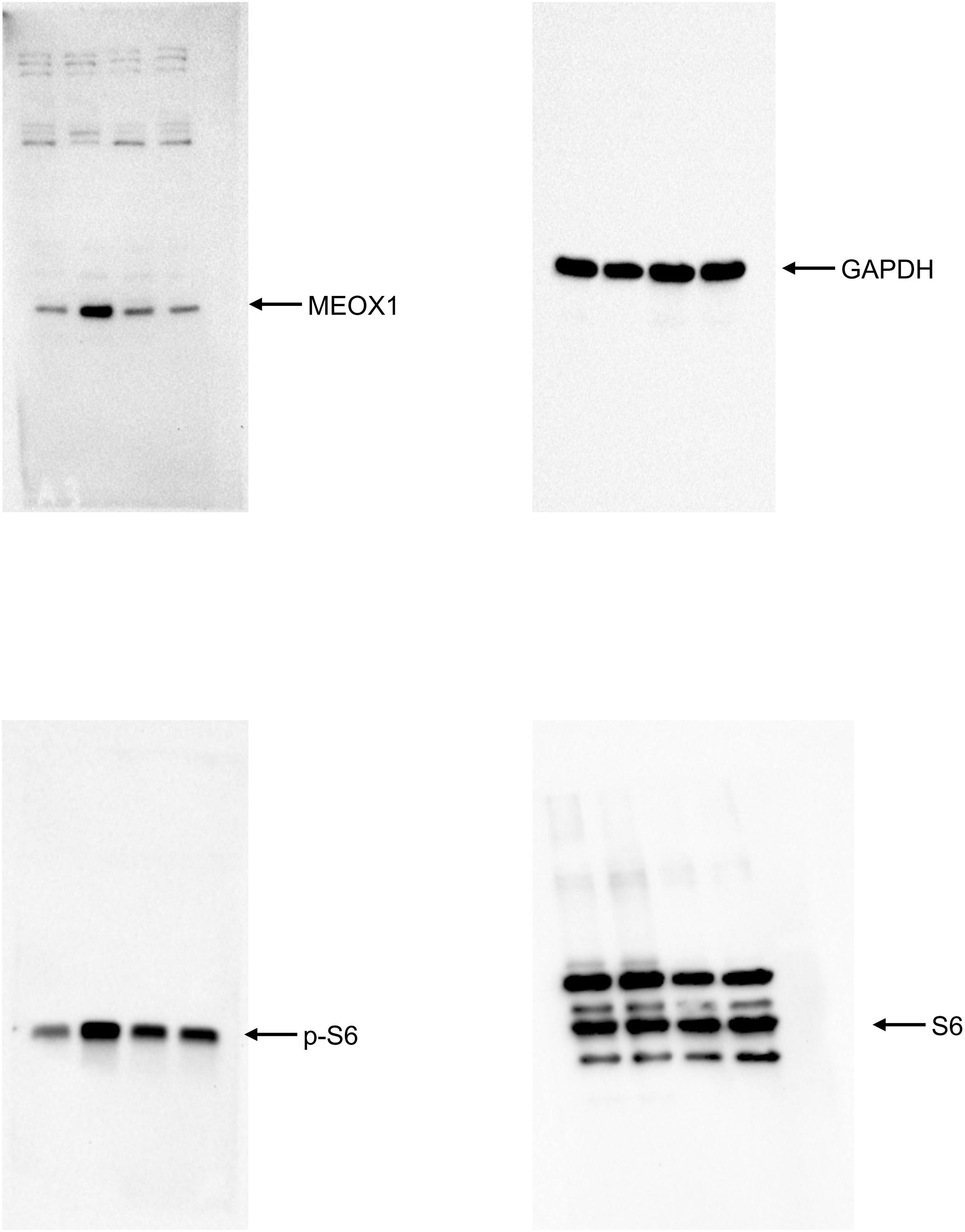

Supplementary Figure 1C

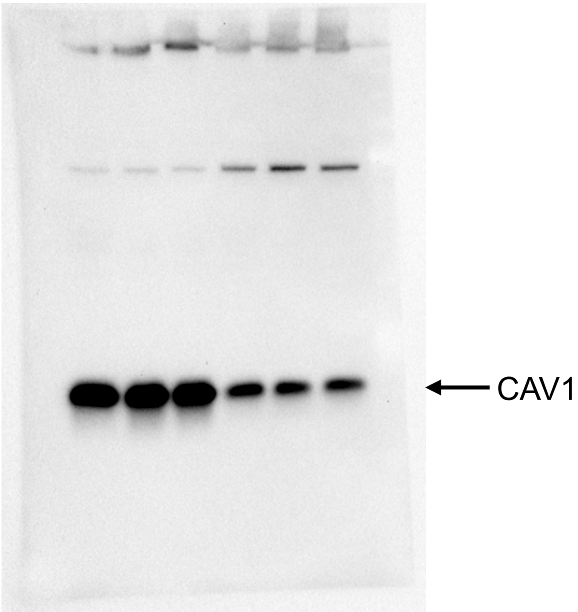

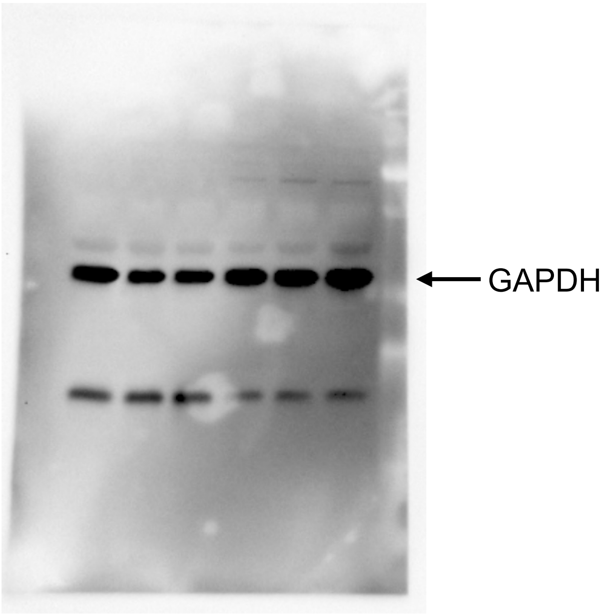

Supplementary Figure 2 E

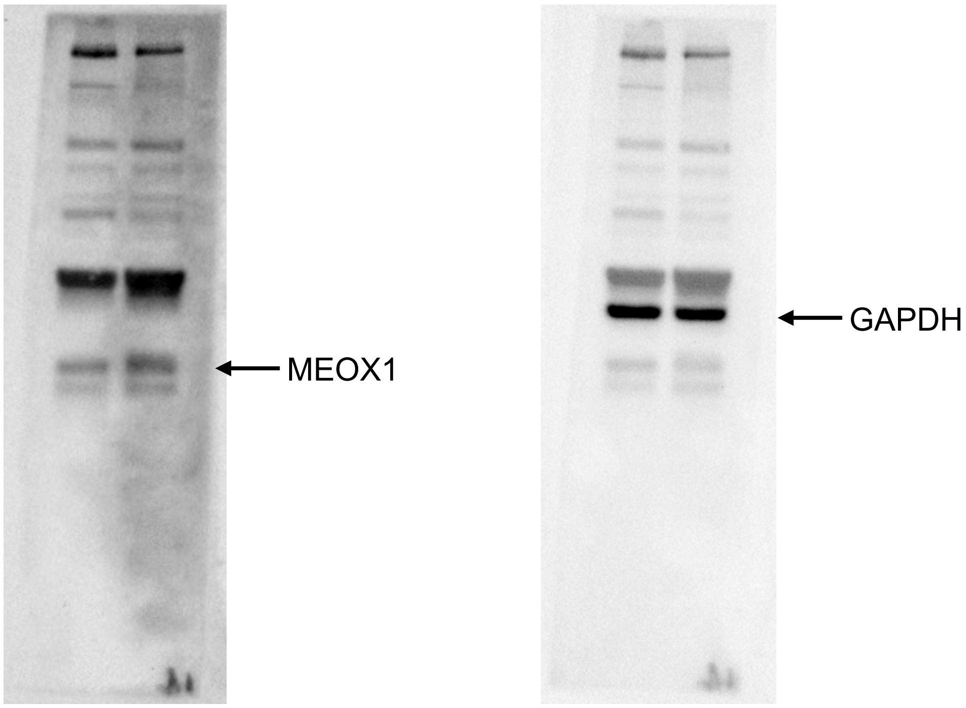

Supplementary Figure 2 F

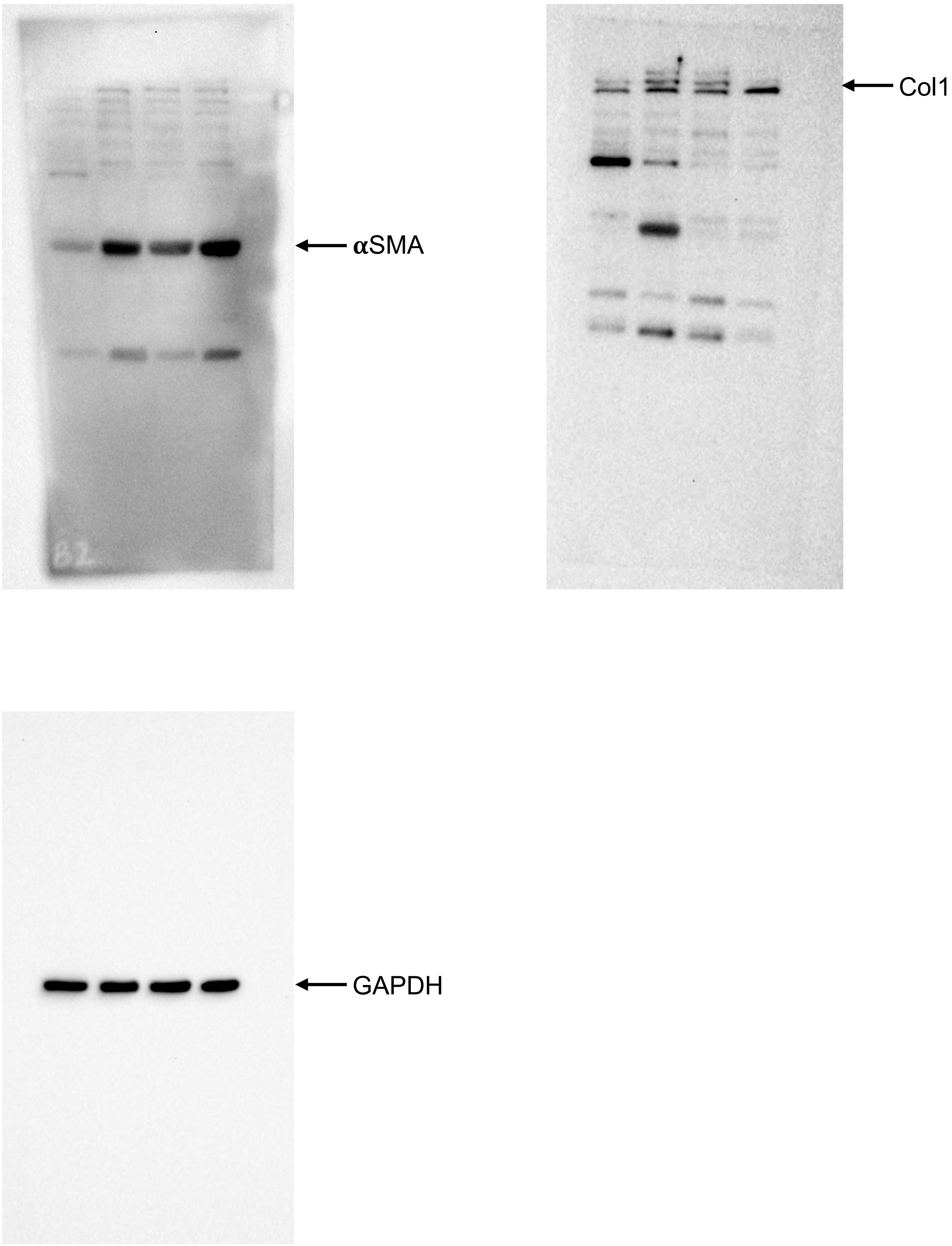

Supplementary Figure 3B

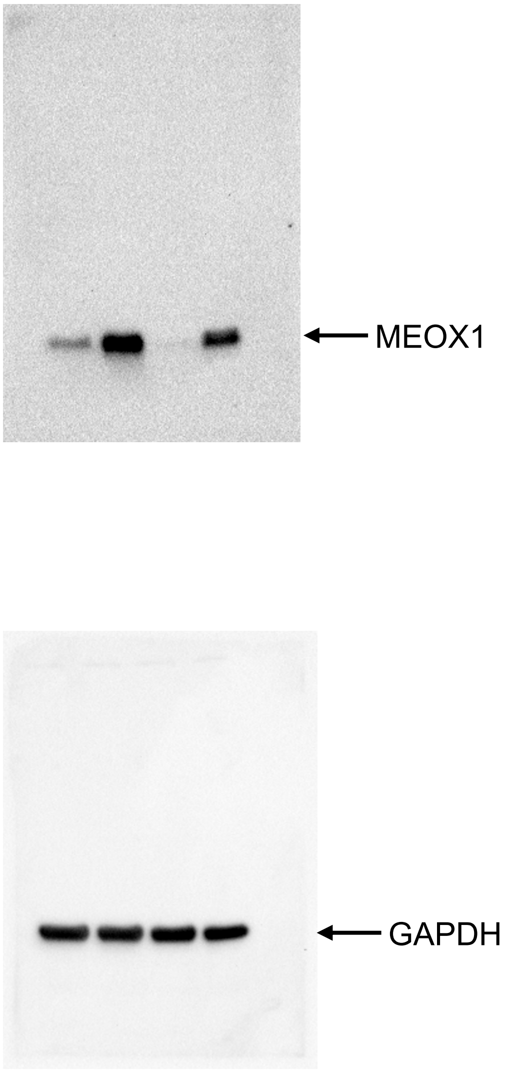

Supplementary Figure 3 C

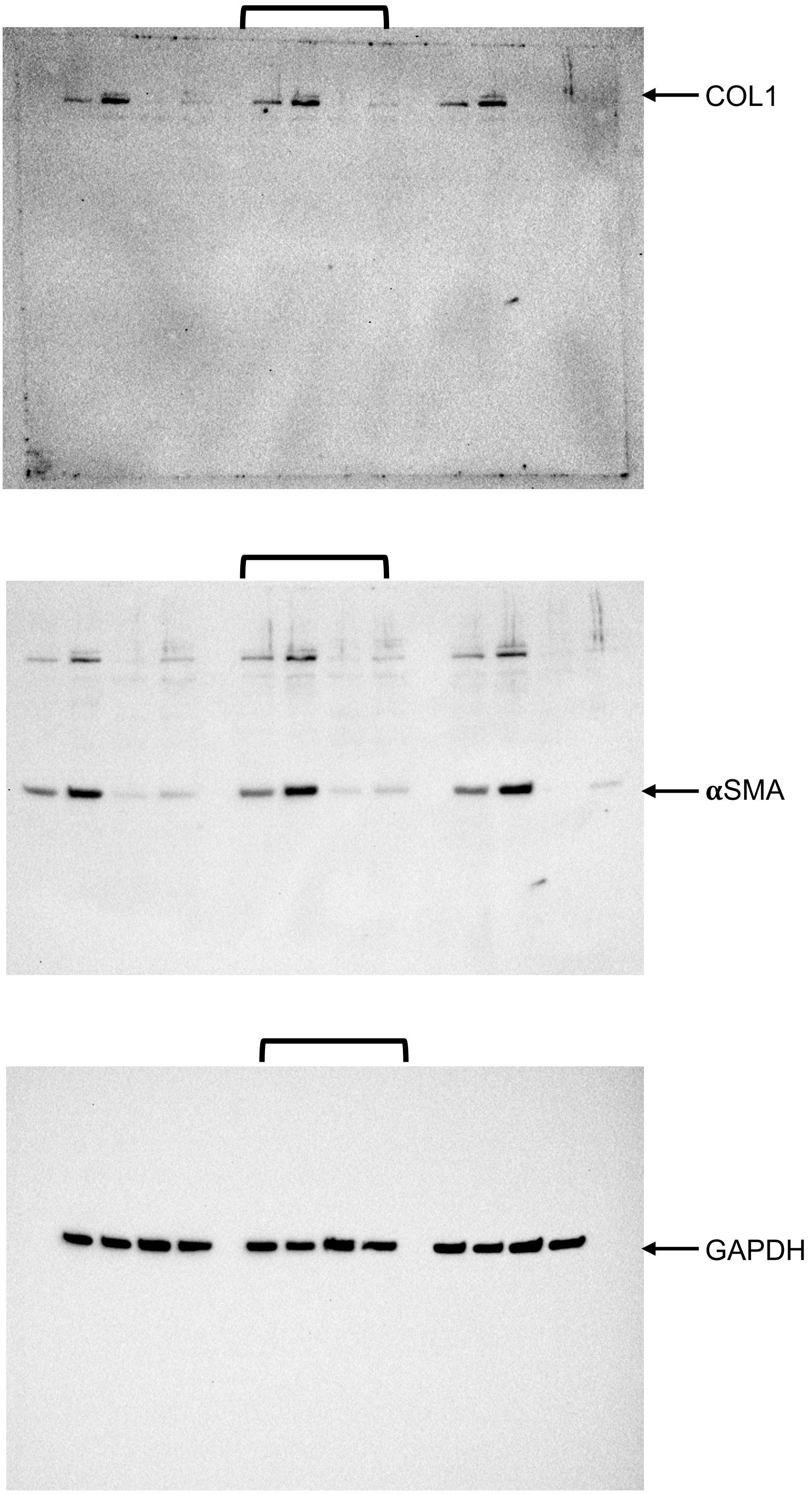

Supplementary Figure 4D

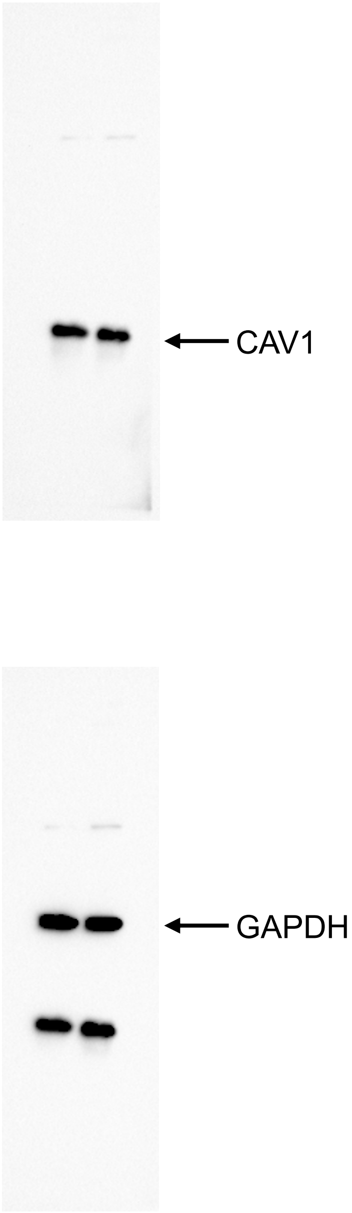

Supplementary Figure 10 A

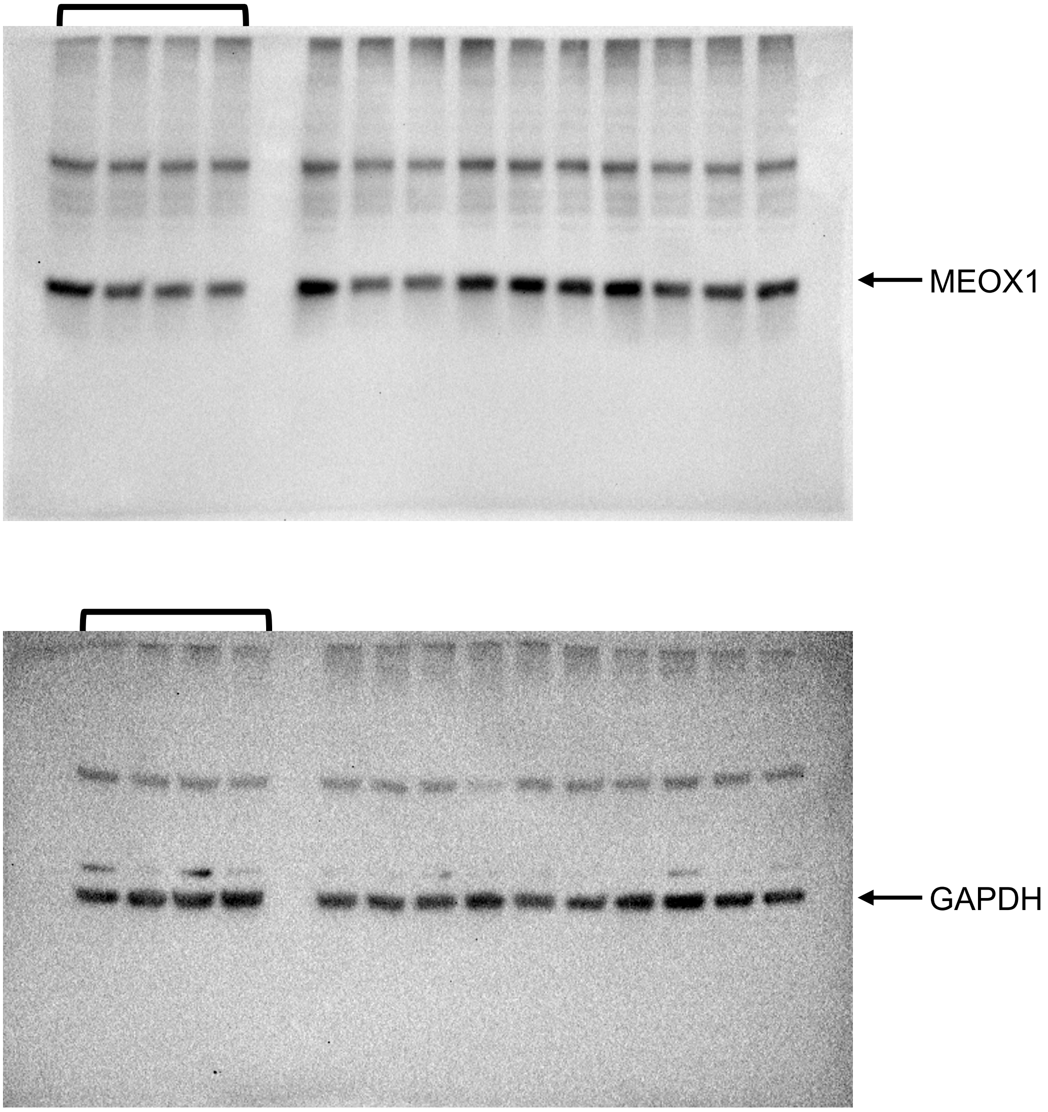

Supplementary Figure 10 B

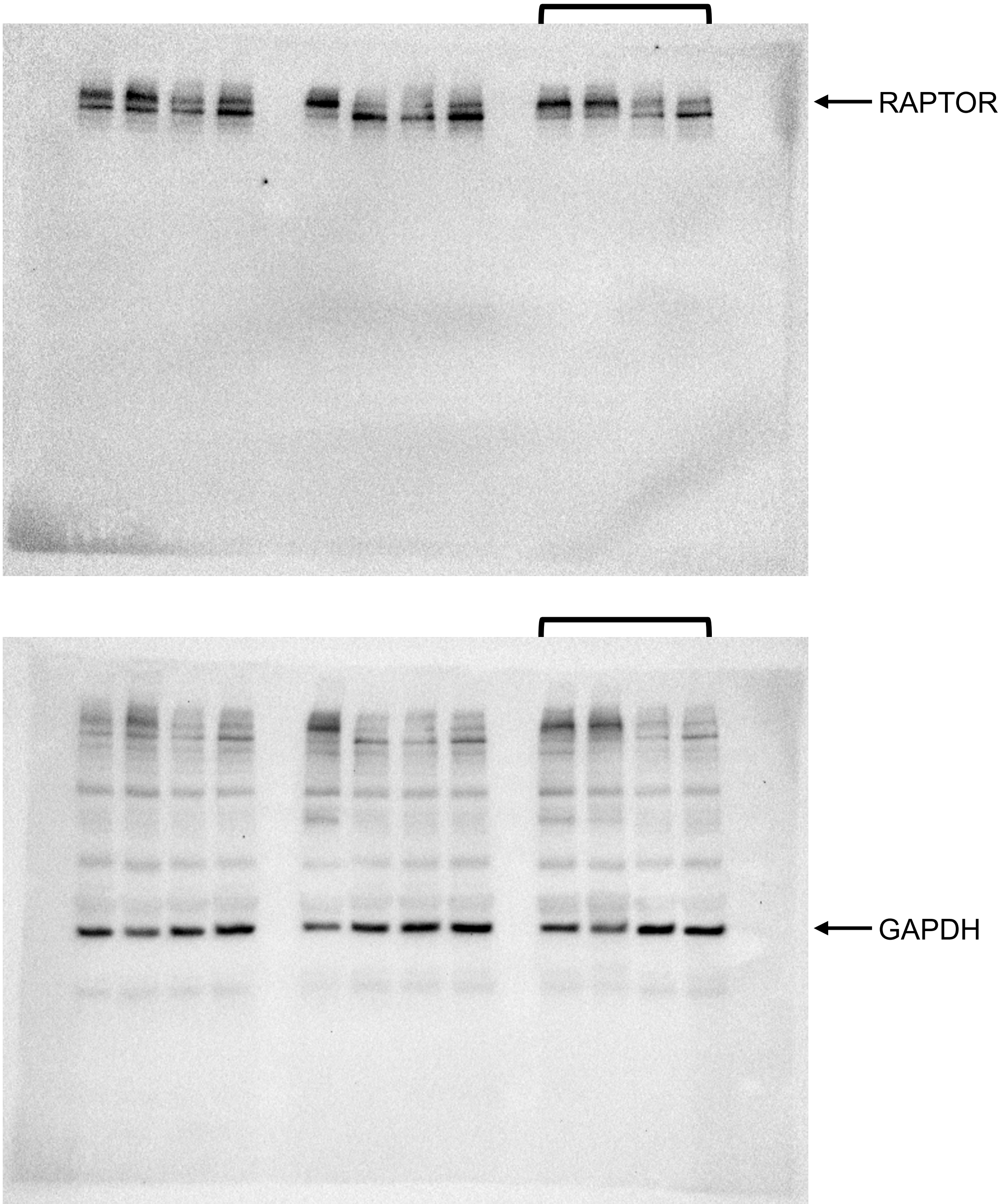

Supplementary Figure 10 C

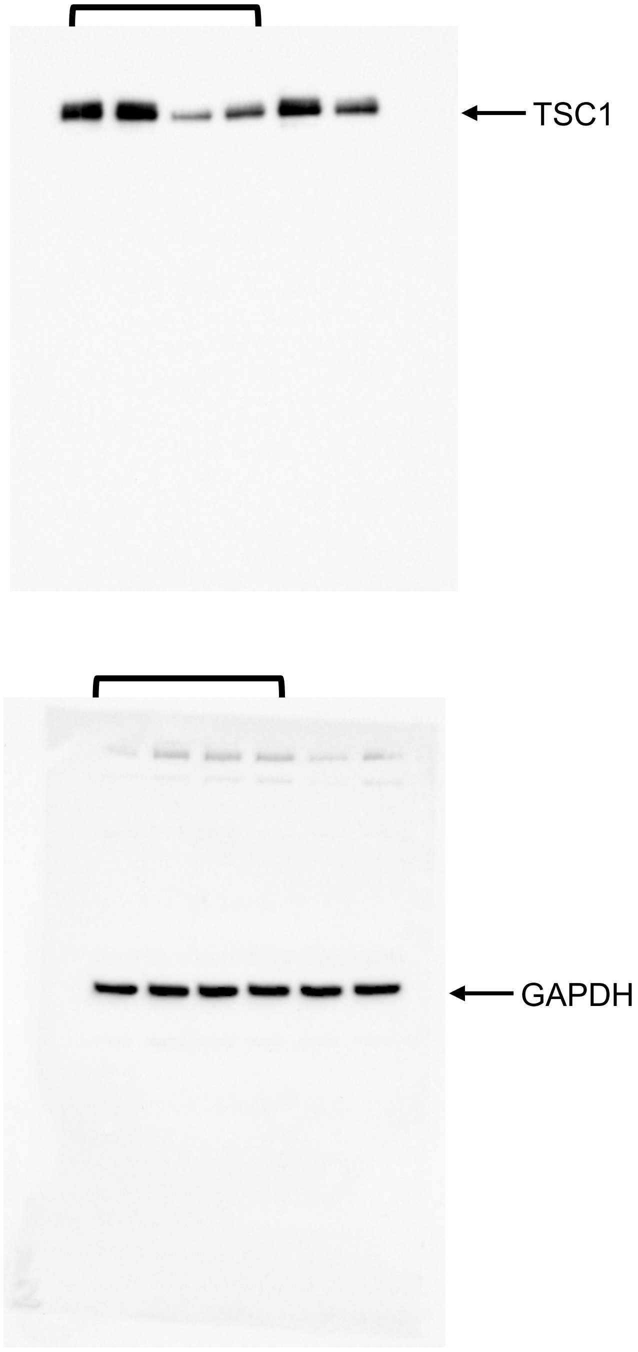

Supplement: Unedited blot and gel images [file jci-136-197651-s146.pdf]
